# Characterization of a Novel Human-Specific STING Agonist that Elicits Antiviral Activity Against Emerging Alphaviruses

**DOI:** 10.1371/journal.ppat.1005324

**Published:** 2015-12-08

**Authors:** Tina M. Sali, Kara M. Pryke, Jinu Abraham, Andrew Liu, Iris Archer, Rebecca Broeckel, Julia A. Staverosky, Jessica L. Smith, Ahmed Al-Shammari, Lisi Amsler, Kayla Sheridan, Aaron Nilsen, Daniel N. Streblow, Victor R. DeFilippis

**Affiliations:** 1 Vaccine and Gene Therapy Institute, Oregon Health and Science University, Portland, Oregon, United States of America; 2 Department of Molecular Microbiology and Immunology, Oregon Health and Science University, Portland, Oregon, United States of America; 3 Iraqi Centre for Cancer and Medical Genetics Research, Baghdad, Iraq; 4 Veterans Affairs Medical Center, Portland, Oregon, United States of America; University of Pennsylvania School of Medicine, UNITED STATES

## Abstract

Pharmacologic stimulation of innate immune processes represents an attractive strategy to achieve multiple therapeutic outcomes including inhibition of virus replication, boosting antitumor immunity, and enhancing vaccine immunogenicity. In light of this we sought to identify small molecules capable of activating the type I interferon (IFN) response by way of the transcription factor IFN regulatory factor 3 (IRF3). A high throughput in vitro screen yielded 4-(2-chloro-6-fluorobenzyl)-N-(furan-2-ylmethyl)-3-oxo-3,4-dihydro-2H-benzo[b][1,4]thiazine-6-carboxamide (referred to herein as G10), which was found to trigger IRF3/IFN-associated transcription in human fibroblasts. Further examination of the cellular response to this molecule revealed expression of multiple IRF3-dependent antiviral effector genes as well as type I and III IFN subtypes. This led to the establishment of a cellular state that prevented replication of emerging Alphavirus species including Chikungunya virus, Venezuelan Equine Encephalitis virus, and Sindbis virus. To define cellular proteins essential to elicitation of the antiviral activity by the compound we employed a reverse genetics approach that utilized genome editing via CRISPR/Cas9 technology. This allowed the identification of IRF3, the IRF3-activating adaptor molecule STING, and the IFN-associated transcription factor STAT1 as required for observed gene induction and antiviral effects. Biochemical analysis indicates that G10 does not bind to STING directly, however. Thus the compound may represent the first synthetic small molecule characterized as an indirect activator of human STING-dependent phenotypes. In vivo stimulation of STING-dependent activity by an unrelated small molecule in a mouse model of Chikungunya virus infection blocked viremia demonstrating that pharmacologic activation of this signaling pathway may represent a feasible strategy for combating emerging Alphaviruses.

## Author Summary

STING is a pattern recognition receptor of cyclic dinucleotides as well as an innate immune adaptor protein that enables signaling from cytoplasmic receptors to the transcription factor interferon regulatory factor 3. Initiation of these pathways leads to the expression of type I interferons and proteins associated with antiviral and antitumor immunity. Small molecules capable of triggering STING-dependent cellular processes are effective at blocking virus replication, enhancing vaccine efficacy, and facilitating immune response to cancer cells. Here we describe the first synthetic small molecule capable of activating STING-mediated signaling in human cells. In addition, we show that exposure of cells to the compound renders them refractory to replication by interferon-sensitive emerging Alphaviruses. In addition, in vivo stimulation of STING-dependent activity also blocks viremia of Chikungunya virus. Ultimately this work may lead to the utilization of STING as a target for multiple immune-mediated therapies.

## Introduction

The innate immune system includes an array of sentinel proteins termed pattern recognition receptors (PRRs) that sense and react to microbe- and danger-associated molecular patterns (reviewed in [1]). These patterns are often constituents or replication intermediates of intracellular (especially viral) pathogens. PRRs respond to this engagement by initiating signaling pathways that bring about the expression or processing of cytokines, chemokines, and effector molecules that both directly block microbial replication and facilitate related adaptive immune processes. As such, PRRs represent an essential first line of immunological defense against infection and are the target of both microbial inhibitory phenotypes as well as pharmacologic manipulation for therapeutic purposes (reviewed in [2]).

Synthesis and secretion of interferon (IFN) proteins is often a primary outcome of PRR-mediated signaling. This includes multiple subtypes of IFNα and β (type I IFN) as well as IFN λ1–3 (type III IFN). IFNs act via cognate cell surface receptors by triggering a phosphorylation cascade involving Janus and tyrosine kinases (Jak1, Tyk2) and signal transducer and activator of transcription 1 and 2 (STAT1/2) transcription factors that amplify the expression of antiviral effector and other immune stimulatory genes conventionally termed IFN-stimulated genes (ISGs). PRR-mediated expression of IFNβ is particularly well characterized and requires phosphorylation of the transcription factor IFN regulatory factor 3 (IRF3) by serine kinases TANK Binding kinase 1 (TBK1) and I Kappa B kinase ε (IKKε) [3]. This occurs primarily through pathways that utilize specific adaptor proteins acting as integration points for upstream PRRs. TIR-domain-containing adaptor-inducing IFNβ (TRIF; also called TICAM1) is required for signals initiated by Toll-like receptors (TLRs) 3 and 4 [4,5]. IFN promoter stimulator 1 (IPS-1; also called MAVS, VISA, Cardif) is employed by RIG-I and MDA5, that both sense cytoplasmic dsRNA [6–9]. Stimulator of IFN genes (STING; also called MITA, TMEM173, MPYS, ERIS) [10–12] is actually both a PRR for cyclic dinucleotides (CDN) via a binding pocket in its C-terminal cytoplasmic domain (CTD) [13–15] as well as an adaptor molecule for multiple cytoplasmic receptors of dsDNA [16–18]. Given the importance of these pathways for innate immune activation and antimicrobial protection they have been the focus of broad and intense research aimed at both understanding their physiological effects and harnessing their potential for contributions to immune-based therapeutics.

Given the ability of the IFN system to render cells and tissues refractory to replication of a wide array of virus types as well as its role in coordinating adaptive immune responses, pharmacologic IFN stimulation has been suggested as a broad spectrum antiviral strategy [2,19–22]. Moreover, factors capable of yielding therapeutic effects via activation of IRF3-mediated responses have been identified and biologically validated. This includes agonists of TLRs shown to block replication of some chronic viruses [23–25] as well as enhance vaccine immunity (reviewed in [26]). Similarly, stimulation of the RIG-I/MDA5/IPS-1 by synthetic nucleic acids can be employed for antiviral outcomes against diverse acute viruses [27,28]. Intriguingly, two synthetic small molecules, 10-carboxymethyl-9-acridanone (CMA) [29] and the chemically unrelated 5,6-dimethylxanthenone-4-acetic acid (DMXAA) [30] are each capable of activating the STING pathway. Both molecules block multiple, even drug-resistant viruses [29,31–33]. Intriguingly, DMXAA exhibits other immunotherapeutic effects including vaccine adjuvanticity [34,35], anti-angiogenic vascular disruption promoting tumor necrosis [36,37], and immune-mediated clearance of solid tumors [38]. Unfortunately, CMA and DMXAA were found to only function in mouse, not human cells and tissues [39–41] and thus were not effective in clinical trials. While analogs of cross-specific stimulatory CDNs have been synthesized [38], to our knowledge there exists no published biological characterization of novel synthetic molecular entities that activate human STING-dependent innate responses, despite the high and multi-pronged therapeutic potential of exploiting this important immunological protein.

Members of the Alphavirus genus include mosquito-transmitted agents that are re-emerging worldwide and can lead to significant morbidity and mortality (reviewed in [42]). Among these is Chikungunya virus (CHIKV), which, despite its evolutionary origin in the Old World, is currently experiencing a severe outbreak in the Caribbean, Central, and South America. Since it first arrived in the Western hemisphere in December 2013 over one million suspected and confirmed cases are estimated to have occurred [43]. CHIKV disease is characterized by severe joint pain that can persist for months to years. Venezuelan Encephalitis virus (VEEV) is a related virus belonging to the New World clade that has experienced numerous outbreaks in South and Central America as well as southern Texas [44]. VEEV is a much more deadly agent with fatality rates at approximately 20% but that can reach up to 35% in children (reviewed in [45]). Currently no FDA-approved antiviral drugs or vaccines exist for either virus. Interestingly, however, both viruses are extremely sensitive to type I IFN [46–48]. Moreover, being RNA-based viruses their infection triggers IRF3/IFN activation via the IPS-1 pathway [49] and as such may not exhibit evasion phenotypes directed at the cytoplasmic DNA-based STING pathway. In light of this pharmacologic activation of IRF3/IFN via STING may represent an efficacious therapeutic strategy. Herein we describe the identification and characterization of a novel small molecule capable of stimulating IRF3 phosphorylation and IFN production in human cells that prevents replication of Alphaviruses. Through reverse genetic studies using CRISPR/Cas9-mediated gene editing we also show that this molecule requires STING for its innate gene induction and antiviral activity and thus it represents the first synthetic compound definitively capable of activating this pathway in human cells. Moreover, in vivo stimulation of the STING pathway was also shown to prevent replication of CHIKV demonstrating the potential therapeutic application of pharmacologically targeting activation of this protein.

## Results

### Identification of a novel IFN/IRF3-inducing molecule by high throughput in vitro screening

**Fig 1:**
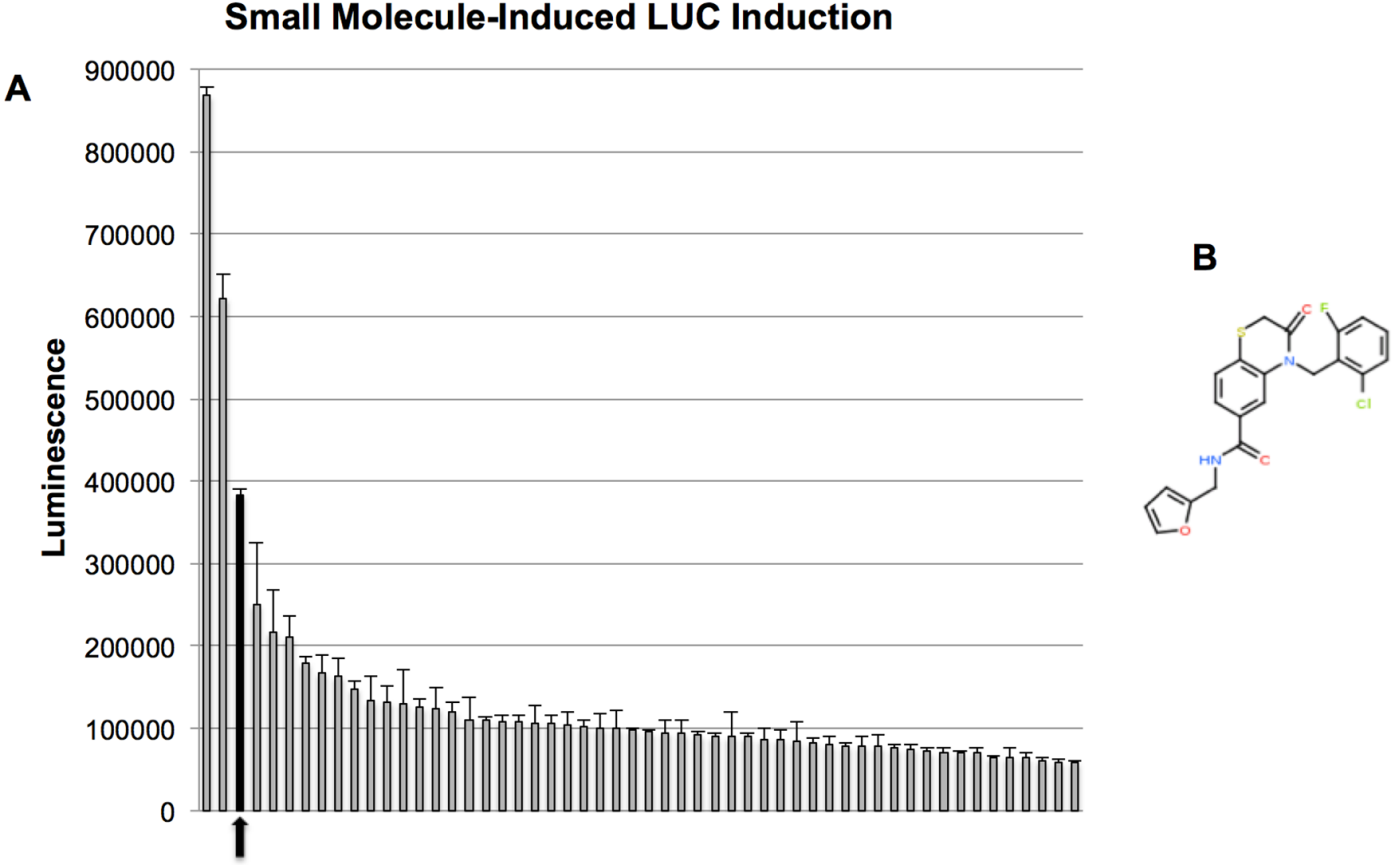
High Throughput Identification of G10. A. Luciferase (LUC) expression of top tier candidate molecules as described in text from a high throughput screen of 51,632 compounds. (A) Presented values represent the average ±SD of duplicate assays. Black bar and arrow indicate LUC signal generated by G10. (B) Chemical structure of 4-(2-chloro-6-fluorobenzyl)-N-(furan-2-ylmethyl)-3-oxo-3,4-dihydro-2H-benzo[b][1,4]thiazine-6-carboxamide (G10).

We sought to discover novel small molecules capable of stimulating innate immune signaling and effector activity in human cells. For this we employed previously described human fibroblasts stably transfected with constitutively expressed human telomerase reverse transcriptase (termed THF) as well as luciferase (LUC) from *Phytonis pyralis* downstream of a promoter element that is reactive to type I IFN-dependent as well as IRF3-dependent transcription (termed THF-ISRE) [18]. Using these cells in a 384-well high-throughput in vitro screening platform we examined 51,632 chemically diverse compounds in duplicate for their ability to significantly stimulate expression of LUC. Sixteen positive control (1000U/mL IFNβ) and negative control (1% DMSO) LUC readings were obtained for each plate (μP and μN averages, respectively). Readings for individual compounds (R) on a single plate were designated as significant if R > (μP-μN)*0.5. Fig 1A illustrates the distribution of raw LUC readings for all molecules that exceeded this threshold on duplicate plates. A compound that exhibited the third highest signal of all those screened was 4-(2-chloro-6-fluorobenzyl)-N-(furan-2-ylmethyl)-3-oxo-3,4-dihydro-2H-benzo[b][1,4]thiazine-6-carboxamide, which we termed G10 (Fig 1B). The two molecules eliciting stronger responses are currently being characterized. As shown in S1 Fig G10 exhibited low cytotoxicity even at high concentrations and was thus selected for more comprehensive examination.

### G10 induces IFN/IRF3- but not NF-κB-dependent transcription in human fibroblasts

**Fig 2:**
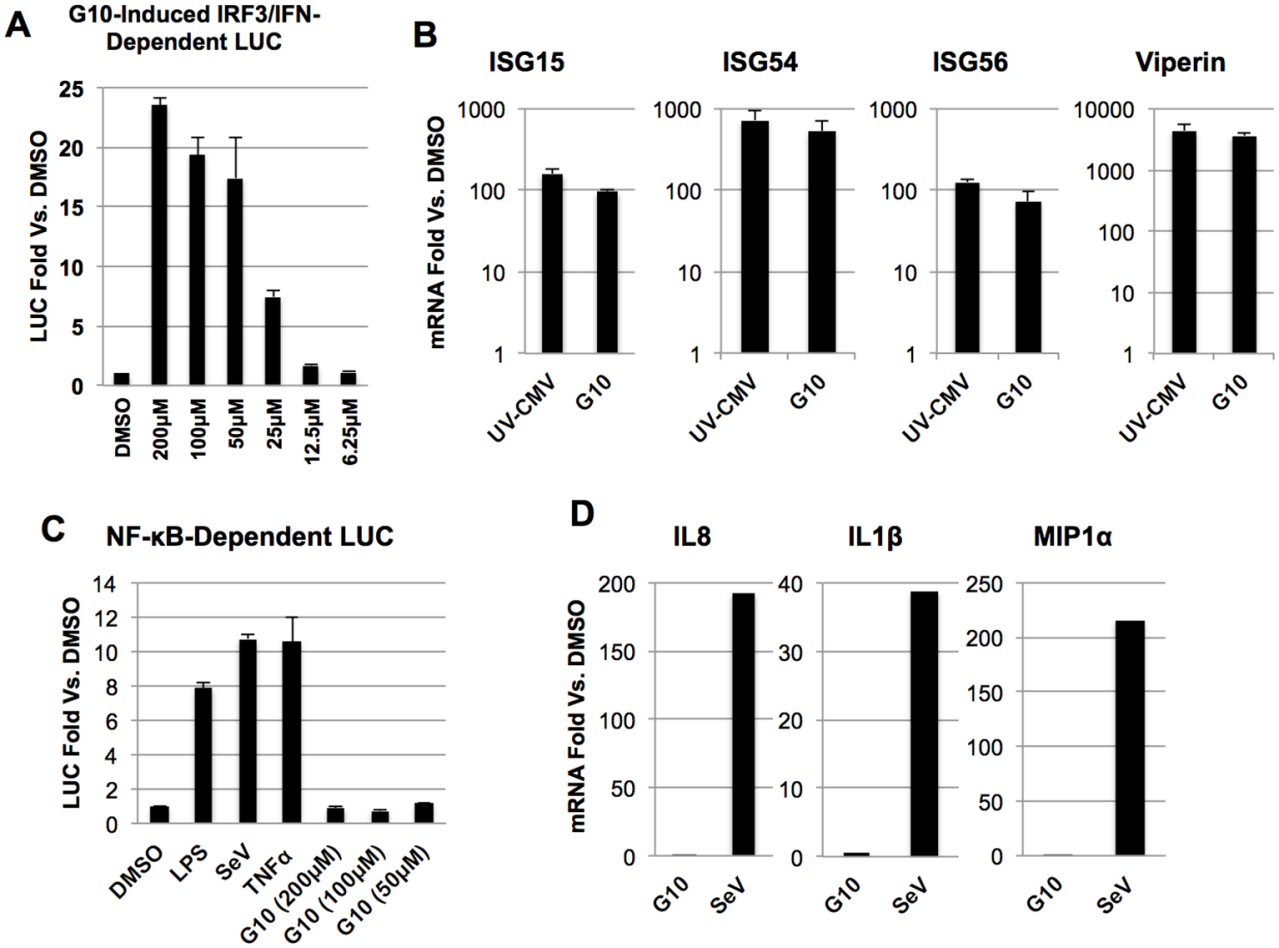
G10 Induces IFN/IRF3- but not NF-κB-Dependent Transcription in Human Fibroblasts. (A) Dose-dependent expression of IRF3/IFN-dependent luciferase (LUC) in telomerized human fibroblasts (THF). Values displayed are average fold changes ±SD of quadruplicate measurements of luminescence following 7h exposure to indicated concentration of G10 relative to cells exposed only to 1% DMSO (all samples normalized to 1% DMSO). (B) mRNA transcription of genes dependent on IRF3/IFN following 7h exposure to 100μM G10 or UV-CMV. Indicated values represent average ±SEM mRNA fold change relative to cells exposed to 1% DMSO from duplicate experiments. (C) Induction of NF-κB-dependent LUC signal in THF reporter cells following 7h exposure to 1μg/mL LPS, 160 HA units/mL SeV, 1ng/mL TNFα or indicated concentration of G10. Values displayed are as described in (A). (D) mRNA transcription of of NF-κB-dependent genes following 7h exposure to 100μM G10 or 160 HA units/mL SeV. Indicated values are mRNA fold change relative to cells exposed to 1% DMSO and are representative of duplicate experiments.

We next validated the G10-mediated induction of IFN/IRF3-dependent LUC in reporter cells by exposing them to a range of concentrations. As shown in Fig 2A, the compound was able to induce LUC expression in a dose-dependent manner. We therefore aimed to verify that the molecule could trigger expression of endogenous genes transcribed in response to IRF3- and IFN-dependent processes. For this we employed semi-quantitative RT-PCR (qPCR) to examine induction of mRNA characterized as dependent on either IRF3 or IFN (ISG54, ISG56, ISG15, Viperin). As shown in Fig 2B, exposing cells to G10 led to transcription of cellular genes triggered by IRF3/IFN-dependent signaling in a manner similar to that induced by exposure to human cytomegalovirus rendered replicationally inactive by UV irradiation (UV-CMV); a stimulus of IRF3 and IFN that occurs via STING and ZBP1/DAI [18,50–52].

Since molecular patterns that culminate in IRF3 activation and synthesis of type I IFN often simultaneously induce pathways leading to activation of the transcription factor NF- κB we next examined whether G10 also stimulated this response. For this we employed telomerized human fibroblasts (THF) stably transduced with LUC driven by an NF-κB-dependent promoter. As shown in Fig 2C we detected no G10-associated stimulation of NF-κB-dependent transcription even when using high concentrations of the compound. This contrasts with what is observed in these cells for other NF-κB-inducing stimuli such as Sendai virus (SeV), TNFα, and LPS. Moreover, the compound also failed to stimulate mRNA synthesis of endogenous NF-κB-dependent genes (IL8, IL1β, and MIP1α Fig 2D). Overall these results suggest that G10 activates IRF3, but not canonical NF-κB pathways in human fibroblasts.

### G10 elicits antiviral activity against New and Old World Alphaviruses

**Fig 3:**
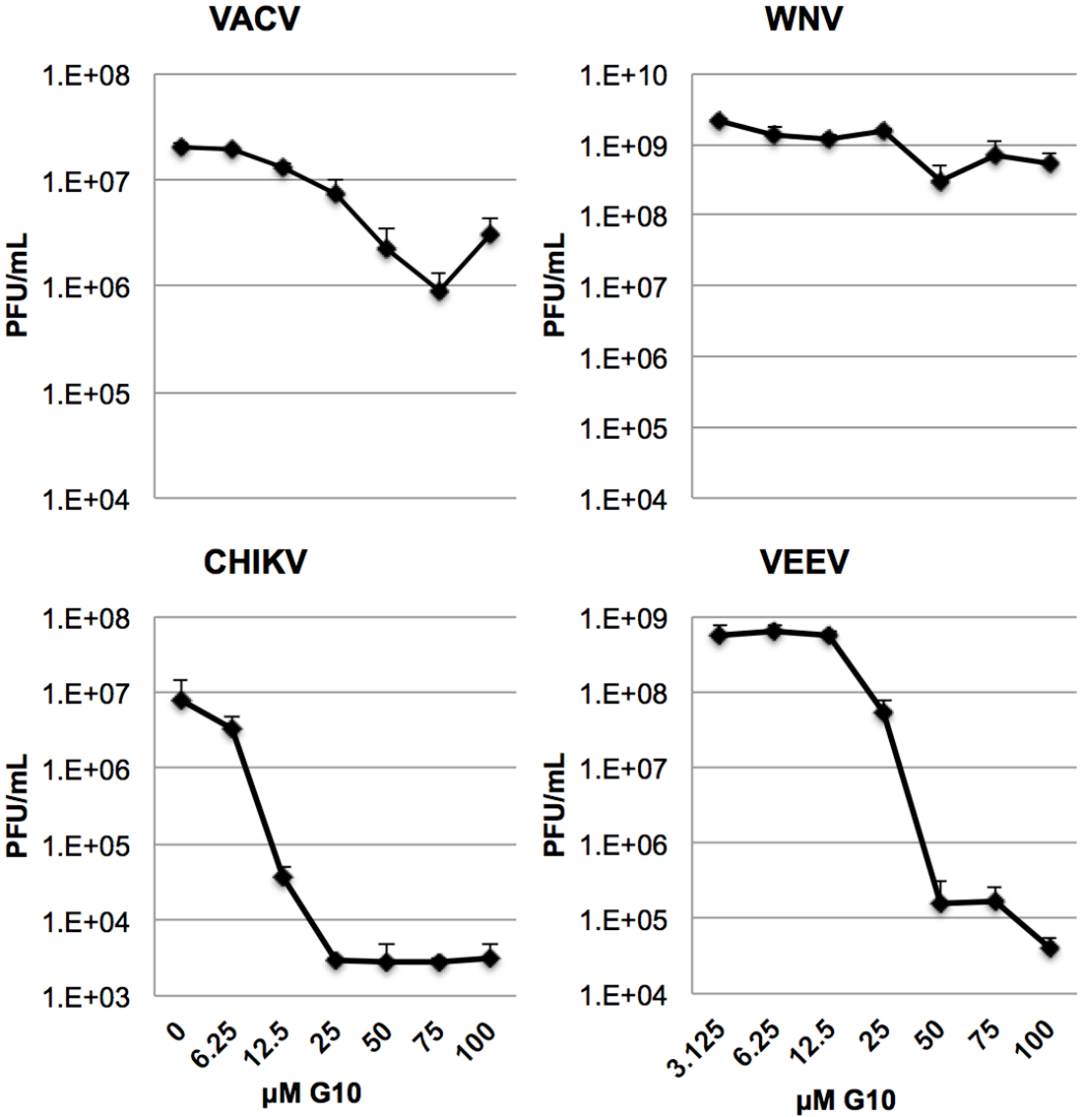
G10 Elicits Antiviral Activity. Average titers ±SD of VACV, WNV, CHIKV, and VEEV grown on THF cells in the presence of indicated G10 concentration (DMSO concentration normalized to 1%). Infections were performed in triplicate and virus harvested at 48h post infection (CHIKV, WNV, CHIKV) or 24h post infection (VACV, VEEV).

Our results indicate that exposure of cells to G10 stimulates the expression of genes that are dependent on IRF3- and/or IFN-dependent signaling. Numerous such genes have been characterized as antiviral effectors that act either via direct molecular or indirect immunological mechanisms (see [53]). We therefore examined whether G10 was correspondingly capable of generating a cellular state refractory to virus replication in vitro, presumably through the initial activity of IRF3. For this we pre-exposed THF cells for 6h to concentrations of G10 that fell well within a nontoxic range (S1 Fig) yet that were observed to trigger innate gene transcription (Fig 2). We next quantitated the replication of West Nile Virus (WNV), Vaccinia Virus (VACV), and Chikungunya virus (CHIKV) on these cells. As shown in Fig 3, the highest concentration of G10 was only able to reduce growth of VACV by approximately one log and WNV by less than one log. Intriguingly, however, the cellular state induced by G10 was much more inhibitory to the growth of CHIKV, reducing replication by over three logs (IC_90_ = 8.01μM; Fig 3). In light of this we examined replication of another clinically relevant Alphavirus species Venezuelan Equine Encephalitis Virus (VEEV). As shown in Fig 3, G10 also potently blocked replication of this virus (IC_90_ = 24.57μM). While related, these species represent highly diverse Alphavirus clades with CHIKV deriving from the Old World and VEEV the New World. Nevertheless G10 was highly effective at similarly impairing replication of both viruses. The relative inability of G10 to render cells as resistant to WNV or VACV replication may reflect the differential susceptibilities of the virus types to the specific antiviral genes induced by G10 or innate evasion phenotypes exhibited by the different viruses (see [54,55]).

### IRF3 is essential to G10-induced transcription and anti-Alphaviral activity

**Fig 4:**
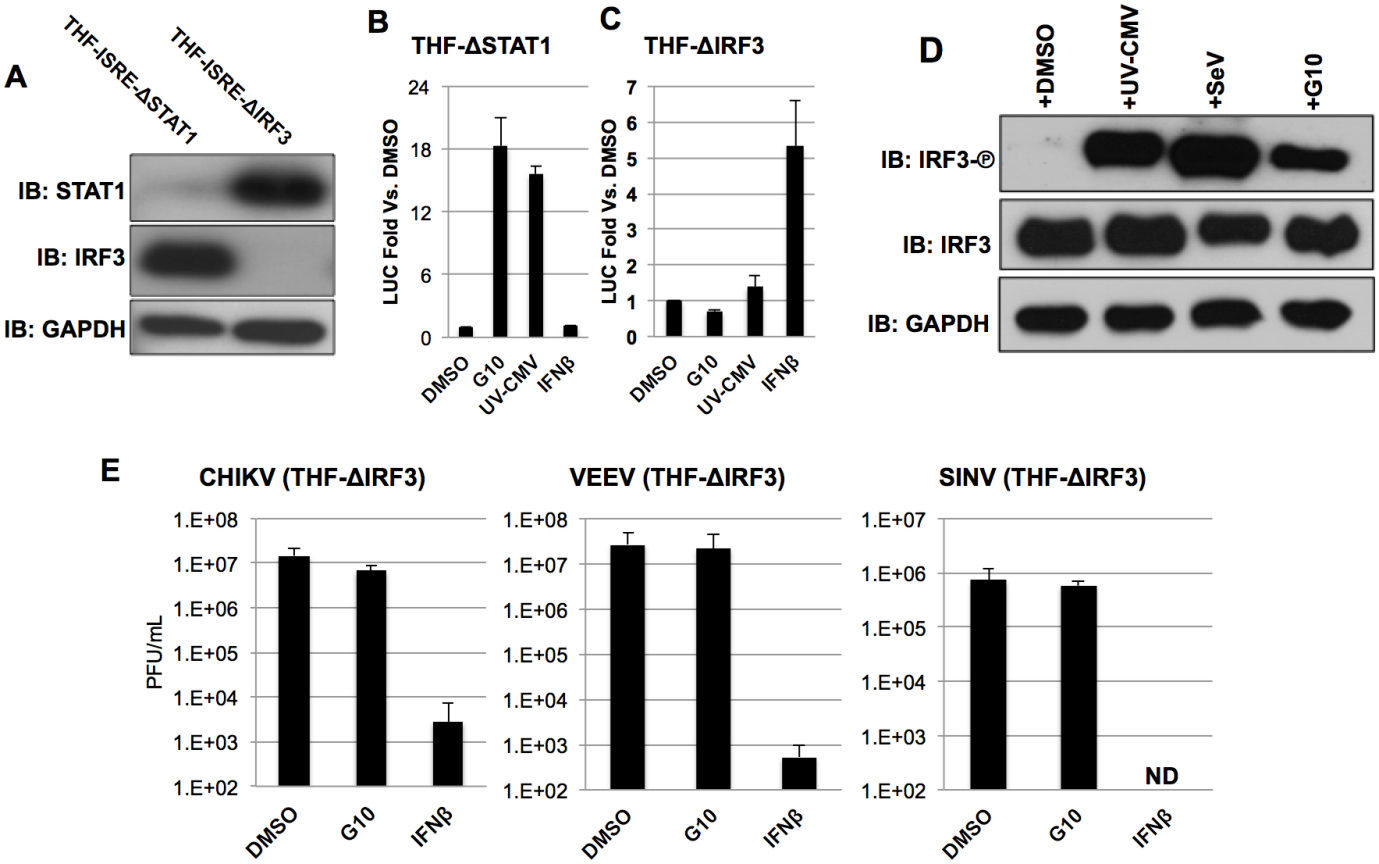
IRF3 is Required for G10-Dependent Transcription and Anti-Alphaviral Activity. (A) Immunoblot showing IRF3, STAT1, and GAPDH in THF-ISRE stably transduced with Cas9 and CRISPR gRNA directed against either STAT1 (THF-ISRE-ΔSTAT1) or IRF3 (THF-ISRE-ΔIRF3) as indicated. (B) Induction of IRF3/IFN-dependent LUC in THF lacking STAT1 following 7h exposure to 100μM G10, UV-inactivated CMV, or 1000U/mL IFNβ. Values displayed are average fold changes ±SD of quadruplicate measurements relative to cells exposed only to 1% DMSO. (C) Induction of IRF3/IFN-dependent LUC in THF lacking IRF3 following 7h exposure to 100μM G10, UV-inactivated CMV, or 1000U/mL IFNβ. Values displayed are as in B. (D) Immunoblot of lysates from THF-ISRE following 6h exposure to DMSO, UV-CMV, SeV or 100uM G10 as indicated showing phosphorylation status of IRF3 S386, total IRF3, and GAPDH. (E) Average media titers +SD of CHIKV, VEEV, and SINV at 24 (VEEV) or 48hpi (CHIKV, SINV) obtained from THF-ISRE-ΔIRF3 cells treated with 1% DMSO, 100μM G10, or 1000U/mL IFNβ as indicated. Infections were performed in triplicate.

Since LUC expression in THF-ISRE reporter cells can be activated directly by IRF3 alone or following IFN-mediated Jak/STAT1/2 signaling we next examined whether either or both transcription complexes were required for this effect. We therefore constructed derivative THF-ISRE cells from which either the STAT1 or IRF3 protein was stably removed via disruption of the respective coding regions by lentivirus-delivered CRISPR/Cas9 components [56–59]. As shown in Fig 4A cells lacking IRF3 (THF-ISRE-ΔIRF3) or STAT1 (THF-ISRE-ΔSTAT1) protein as detectable by immunoblot (IB) were obtained. Absence of these proteins was functionally validated by the elimination of STAT1- (Fig 4B) or IRF3-dependent (Fig 4C) LUC expression following treatment with control stimuli IFNβ and UV-inactivated cytomegalovirus (UV-CMV), respectively. Using these cells, G10 was found to induce LUC expression in the absence of STAT1. However, G10-induced LUC expression was abrogated in cells lacking IRF3. These results suggest that the innate immune stimulation observed in response to G10 is dependent on the IRF3 transcription factor and does not require direct activation of Jak/STAT-dependent signaling.

Since transcription of a reporter gene by G10 is abolished in the absence of IRF3 this strongly implies that G10 stimulates the activation of IRF3, which involves phosphorylation of C-terminal serine residues and subsequently allows its dimerization, nuclear translocation and DNA binding. To verify that IRF3 activation does occur in response to G10 we performed IB using an antibody reactive to phosphorylated IRF3 residue S386 with whole cell lysates harvested from THF exposed to G10 or control stimuli. As shown in Fig 4D G10 stimulates phosphorylation of IRF3 in a manner similar to that triggered by UV-CMV and SeV. We next examined whether IRF3 was involved in establishment of the observed G10-mediated anti-Alphaviral state (Fig 3). To answer this we utilized THF-ISRE-ΔIRF3 by exposing them to DMSO, 100μM G10 (a concentration over twelve times the IC_90_ for CHIKV and over four times the IC_90_ for VEEV), or 1000U/mL IFNβ and examined growth of CHIKV and VEEV after a period that enables peak viral titers. Fig 4E shows that while a strong anti-Alphaviral state can still be established in these cells by pre-exposure to IFNβ, the ability of G10 to block replication of CHIKV and VEEV is lost. In addition, we also examined Sindbis virus (SINV), another Old World Alphavirus species, that grows poorly on wild type human fibroblasts (S2 Fig) but can replicate when the IPS-1-IRF3-IFN response is impaired. As shown in Fig 4E cells lacking IRF3 are permissive for SINV replication. However, in these cells IFNβ, but not G10, is capable of inhibiting virus replication. These results indicate the anti-Alphaviral activity elicited by G10 requires IRF3-dependent cellular responses.

### G10-mediated IRF3 activation and anti-Alphaviral activity occur independently of IPS-1/MAVS-dependent signaling

**Fig 5:**
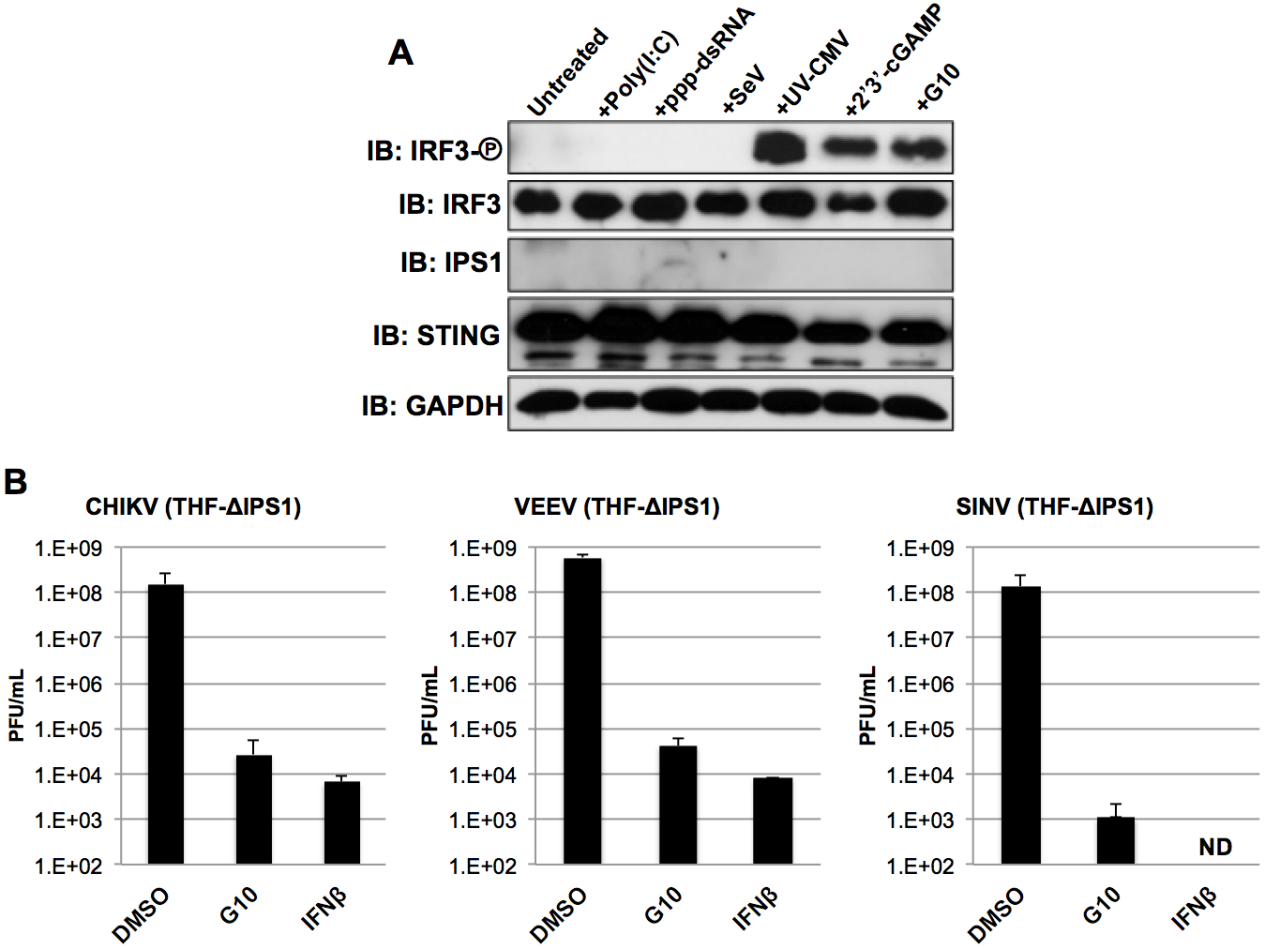
G10 Elicits IRF3 phosphorylation and Anti-Alphaviral Activity in Cells Lacking IPS1. (A) Immunoblot of lysates from THF-ISRE-ΔIPS1 following 6h exposure to DMSO, UV-CMV, SeV or 100uM G10 as indicated showing phosphorylation status of IRF3 S386, total IRF3, IPS1, STING, and GAPDH. (B) Average media titers +SD of CHIKV, VEEV, and SINV at 24h (VEEV) or 48h (CHIKV, SINV) post infection obtained from THF-ISRE-ΔIPS1 cells treated with 1% DMSO, 100μM G10, or 1000U/mL IFNβ as indicated. Infections were performed in triplicate.

An array of PRRs reacting with multiple classes of pathogen-associated molecules is capable of initiating signaling pathways that terminate in IRF3 activation. As discussed above these conventionally employ an adaptor protein to activate the IRF3-directed kinases TBK1 and IKKε. IFNβ promoter stimulator 1 (IPS-1, also called MAVS) is utilized by RIG-I and MDA5, cytoplasmic sensors of (typically virus-associated) dsRNA. In an effort to characterize the cellular pathway targeted by G10 we first asked whether IPS-1 is important for the molecule’s effect on innate cellular activation. To address this we again employed lentivirus—delivered CRISPR/Cas9 to construct THF cells lacking the protein. As shown in Fig 5A disruption of the IPS-1 coding region in this manner correspondingly results in undetectable protein. Furthermore, IRF3 activation in response to poly(I:C), ppp-dsRNA, or SeV infection, processes requiring IPS-1 [8,60,61], are accordingly abrogated in these cells. In contrast, IRF3 activation by UV-CMV or 2’3’-cGAMP, which occur via the STING pathway in these cells [13,52,62] and are independent of IPS-1 remains intact. Likewise, G10-induced IRF3 phosphorylation is also functional in these cells (Fig 5A) indicating that the molecule stimulates a response that does not require components of the IPS-1 signaling apparatus. We next asked whether the anti-Alphaviral activity elicited by G10 similarly occurred independently of IPS-1 as described above. As shown in Fig 5B, pretreatment of cells with either G10 or IFNβ diminishes replication of all three virus types by multiple logs; a magnitude that parallels what is observed in wild type cells (Fig 3). Together these results indicate that the IRF3-dependent anti-Alphaviral activity conferred by G10 does not require IPS-1 or, by extension, any upstream PRRs (i.e. RIG-I, MDA5, POL3) associated with this signaling pathway.

### STING is required for G10-mediated IRF3 activation, gene expression, and anti-Alphaviral activity

**Fig 6:**
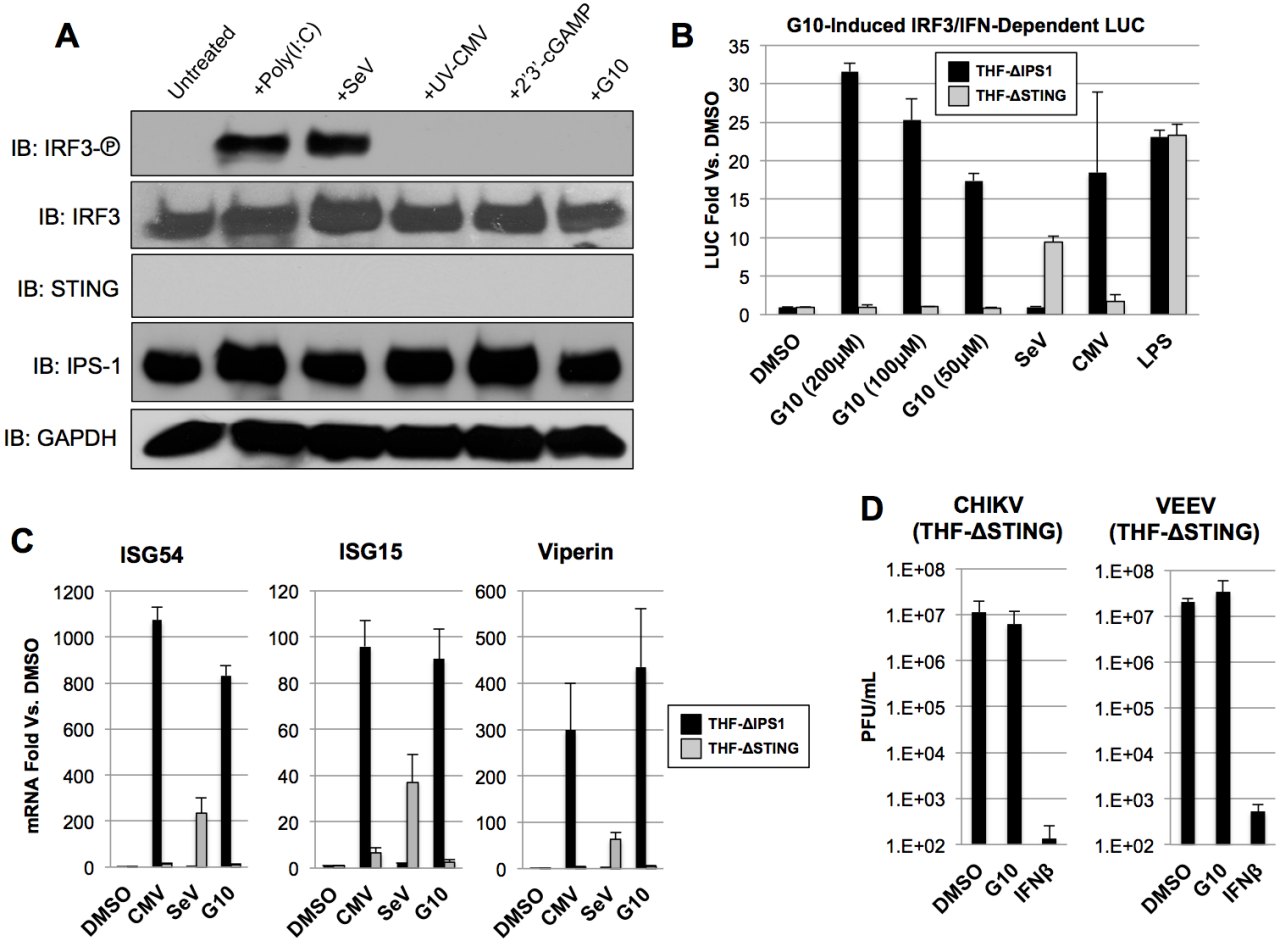
G10-Mediated IRF3 Phosphorylation, ISG mRNA Induction, and Anti-Alphaviral Activity are not Detectable in Cells Lacking STING. (A) Immunoblot of lysates from THF-ΔSTING following 7h exposure to 1% DMSO, 0.1μg/mL poly(I:C), SeV, UV-CMV, 1μg/mL 2’3’-cGAMP, or 100uM G10 as indicated showing phosphorylation status of IRF3 S386, total IRF3, IPS1, STING, and GAPDH. (B) Expression of IRF3/IFN-dependent LUC in THF-ISRE-ΔIPS1 and THF-ISRE-ΔSTING following 7h exposure to 1% DMSO, indicated concentrations of G10, SeV, UV-CMV, or 1μg/mL LPS. Values are presented as average fold change in quadruplicate measurements ±SD relative to cells treated with 1% DMSO. (C) Average fold changes ±SD from duplicate experiments of ISG54, ISG15, and Viperin mRNA relative to cells treated with 1% DMSO in THF-ISRE-ΔIPS1 (black bars) or THF-ISRE-ΔSTING (gray bars) following exposure to UV-CMV, SeV, or 100μM G10. (D) Media titers of CHIKV and VEEV at 24h (VEEV) or 48h (CHIKV) obtained from THF-ISRE-ΔSTING cells treated with 1% DMSO, 100μM G10, or 1000U/mL IFNβ as indicated. Infections were performed in triplicate.

We next examined whether the IRF3-terminal adaptor protein STING is critical to G10-mediated innate activation. For this we constructed THF-ISRE cells from which the STING protein is eliminated via CRISPR/Cas9-mediated gene disruption as described above. Knockout of the protein was confirmed visually by IB of whole cell lysates and functionally by demonstrating the absence of IRF3 S386 phosphorylation following treatment with UV-CMV or transfection with 2’3’-cGAMP, both of which are STING-dependent cellular reactions (Fig 6A). IPS-1-dependent signaling remains operational in these cells, however, as evidenced by IRF3 phosphorylation in response to SeV exposure and poly(I:C) transfection. Intriguingly, STING deletion resulted in elimination of G10-induced IRF3 S386 phosphorylation. We next examined whether the G10-induced transcriptional response observed in wild type cells (Fig 2) was similarly inactive in STING-deficient cells. For this we also included THF-ISRE-ΔIPS-1 cells as a control for any potential off-target effects of CRISPR/Cas9 genome editing or lentivirus transduction. As shown in Fig 6B, treatment with G10 leads to strong LUC induction in cells lacking IPS-1, as does exposure to UV-CMV or LPS (a TRIF-dependent process) but not SeV. However, in the absence of STING both G10 and UV-CMV fail to activate appreciable LUC whereas SeV and LPS induce substantial expression thus validating intact IRF3-terminal signaling. Transcriptional induction of IRF3-dependent endogenous host genes was also examined by qPCR. Fig 6C shows that, similar to LUC expression, ISG54, ISG15, and Viperin mRNAs are highly transcribed in response to UV-CMV and G10 exposure in cells lacking IPS-1 but remain comparatively unstimulated in cells lacking STING. In contrast, SeV-induced transcription of these genes is only observed in cells lacking STING. From these results we conclude that the IRF3 activation and IRF3-dependent transcriptional activity in response to G10 occurs via a STING-dependent pathway.

Based on our observations above that G10-elicited anti-Alphaviral activity requires IRF3 (Fig 4), we hypothesized that STING-dependent IRF3 signaling is also essential to this process. We therefore examined replication of CHIKV and VEEV in THF-ISRE-ΔSTING cells following treatment with DMSO, G10, or IFNβ as described above (these cells are not permissive for SINV replication since IPS-1-IRF3-IFN signaling is intact [49]). As shown in Fig 6D the ability of G10 to block virus replication is absent in cells lacking STING despite the fact that an antiviral state can effectively be established as indicated by treatment with IFNβ. Overall these data clearly indicate that the STING pathway is crucial to the IRF3-dependent innate antiviral activity induced by G10.

STING behaves as a PRR of cyclic dinucleotides (CDN) by way of a direct interaction between them and the protein’s C-terminal (ligand-binding) domain [13,63]. We therefore asked whether G10 was a direct ligand of human STING, similar to the mouse STING-specific small molecules DMXAA [40,41,64,65] and CMA [39], and sought evidence supporting this hypothesis. For this we used differential scanning fluorimetry to examine changes in thermal stability of purified STING-CTD in the presence of G10. Thermal stability of the protein is expected to increase with binding affinity of protein-ligand complexes [66,67]. However, as shown in S3 Fig, incubating purified mouse or human STING-CTD with G10 did not increase the protein’s thermal stability, as does a validated ligand such as 2’3’-cGAMP or DMXAA (in the case of mouse protein). These observations are inconsistent with G10 binding directly to human or mouse STING-CTD.

STING also behaves as an adaptor molecule [68] required for activating IRF3-targeting kinases by multiple upstream cytoplasmic DNA-sensing PRRs including ZBP1/DAI [69], IFI16 [16], DDX41 [70], and IFI203 [17]. Given that we were unable to detect evidence of direct interaction with STING it is possible that G10 engages one or more of these (or an as yet unknown; see [71]) PRRs to initiate STING-dependent activity. In light of this it is interesting to note that G10 does not induce IRF3 activation or IRF3-dependent gene expression in the immortalized promonocytic cell line THP-1 despite the fact that these cells express phenotypically active STING [16,70,72–77] (S4 Fig). These results are potentially informative with respect to identity of the direct cellular target of G10 since it likely rules out cGAS [14], DDX41 [73], and IFI16 [16] based on the observation that these are present and functional in THP-1 cells [78]. Currently we are examining whether other known STING-dependent receptors (IFI203 and ZBP1/DAI) are required for G10-mediated activity.

### G10 induces STING-dependent synthesis and secretion of bioactive interferon

**Fig 7:**
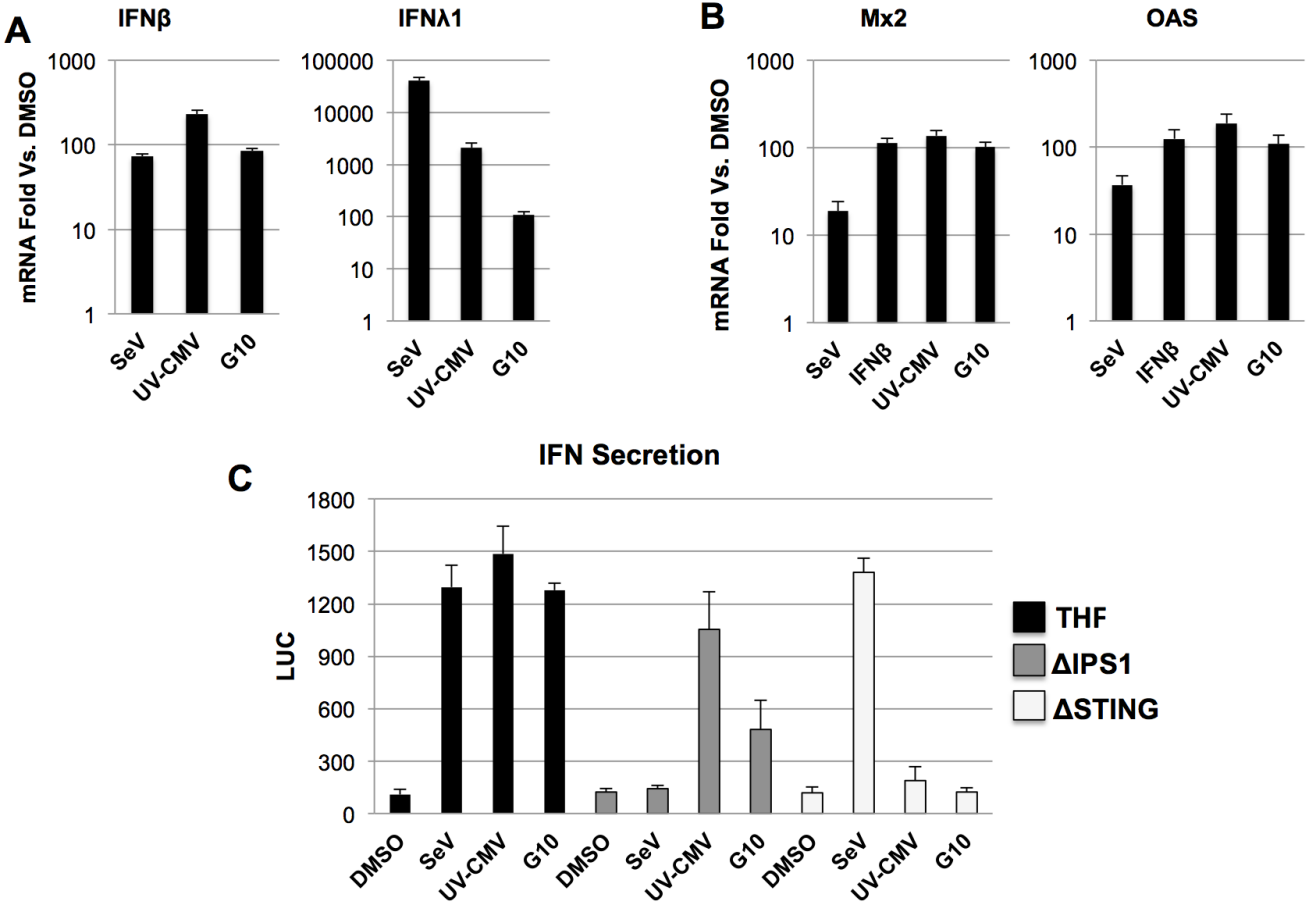
G10-Mediated Expression of Type I and III IFN and IFN-Dependent Genes. Induction of IFNβ and IFNλ1 (**A**) or Mx2 and OAS (**B**) transcripts in THF following 18h exposure to SeV, UV-CMV, or 100μM G10 or IFNβ as indicated. Values presented are average fold changes relative to cells treated with 1% DMSO ±SD based on duplicate treatments. (C) Secretion of type I IFN from THF-ISRE, THF-ISRE-ΔIPS1, or THF-ISRE-ΔSTING following 18h exposure to 1% DMSO, SeV, UV-CMV, or 100μM G10. Average LUC values ±SD were obtained from THF-ISRE-ΔIRF3 exposed (in triplicate) to media harvested from indicated cells and exposed to indicated stimulus (in triplicate).

Our data indicate that G10 induces expression of cellular antiviral effector genes and that this process ultimately requires IRF3 and STING. However, transcription of these genes (ISG15, Viperin, ISG54, ISG56) can be triggered in response to either activated IRF3 or IFN-dependent (Jak/STAT) signaling [79–81]. Therefore, we next aimed to discern the respective roles of these pathways in G10-mediated innate and antiviral activation. For this we first examined whether G10 is able to stimulate expression of type I or III interferons, both of which are known to induce Jak/STAT-dependent signaling via type I and type III IFN receptor complexes, respectively. As shown in Fig 7A, exposure of THF-ISRE to G10 leads to transcription of both IFNβ and IFNλ1 mRNAs. However, induction of IFNλ1 by G10 does not approach the levels observed for UV-CMV and SeV suggesting the involvement of different, potentially G10-independent, cellular factors in this process. We next examined whether genes known to be induced solely by Jak/STAT- (as opposed to either Jak/STAT or IRF3) dependent signaling were also induced following G10 exposure since this would be indicative of autocrine/paracrine signaling following release of type I or type III IFN from treated cells. In contrast to the 7h treatments used above (Figs 2 and 6) we exposed cells to indicated stimuli for 18h in order to allow for completion of the full sequence of IFN transcription, synthesis, secretion, and autocrine/paracrine signal transduction. Fig 7B illustrates substantial induction of known IFNα/β-dependent genes Mx2 and OAS in response to exposure to G10 as well as other control stimuli including IFNβ. These results are consistent with the secretion of IFN in response to treatment with G10.

Numerous subtypes of type I and III interferons exist and thus demonstrating the presence of secreted molecules requires type-specific immunoassays (ELISA). We chose to examine secreted interferon of all subtypes by employing a cell-based reporter assay that reacts with any bioactive type I or III interferon species. For this we utilized THF-ISRE-ΔIRF3 cells described above (Fig 4). Since these cells cannot make type I IFN (due to the absence of IRF3) they do not generate an autocrine LUC reporter signal in response to exogenous IFN-inducing stimuli and only react to IFN itself (Fig 4). We treated THF-ISRE, THF-ISRE-ΔIPS-1, and THF-ISRE-ΔSTING with DMSO, SeV, UV-CMV, or G10 for 18h and transferred media from these cells to THF-ISRE-ΔIRF3 for 8h. Next, IFN-dependent luciferase expression was measured by luminescence. In agreement with our previous observations, IFN secretion was detected by wild type THF cells exposed to UV-CMV, SeV, or G10. In cells lacking IPS-1 secretion of IFN was abolished in response to SeV but not UV-CMV or G10. Moreover, cells lacking STING failed to secrete IFN in response to UV-CMV or G10 but SeV-induced secretion was intact. Interestingly, G10-induced secretion by cells lacking IPS-1 was significantly diminished relative to wild type cells (p = 0.00013) perhaps indicating pivotal crosstalk between STING and IPS-1 signaling, a phenomenon previously alluded to [11]. However, as indicated by Fig 6 the role of IPS-1 does not appear to be essential for G10’s anti-Alphaviral activity. Overall these data indicate that G10 triggers the STING-dependent transcription, synthesis, and secretion of IFN species capable of initiating Jak/STAT signaling and ISG expression.

### STAT1 is required for G10-mediated anti-Alphaviral activity and ISG expression

**Fig 8:**
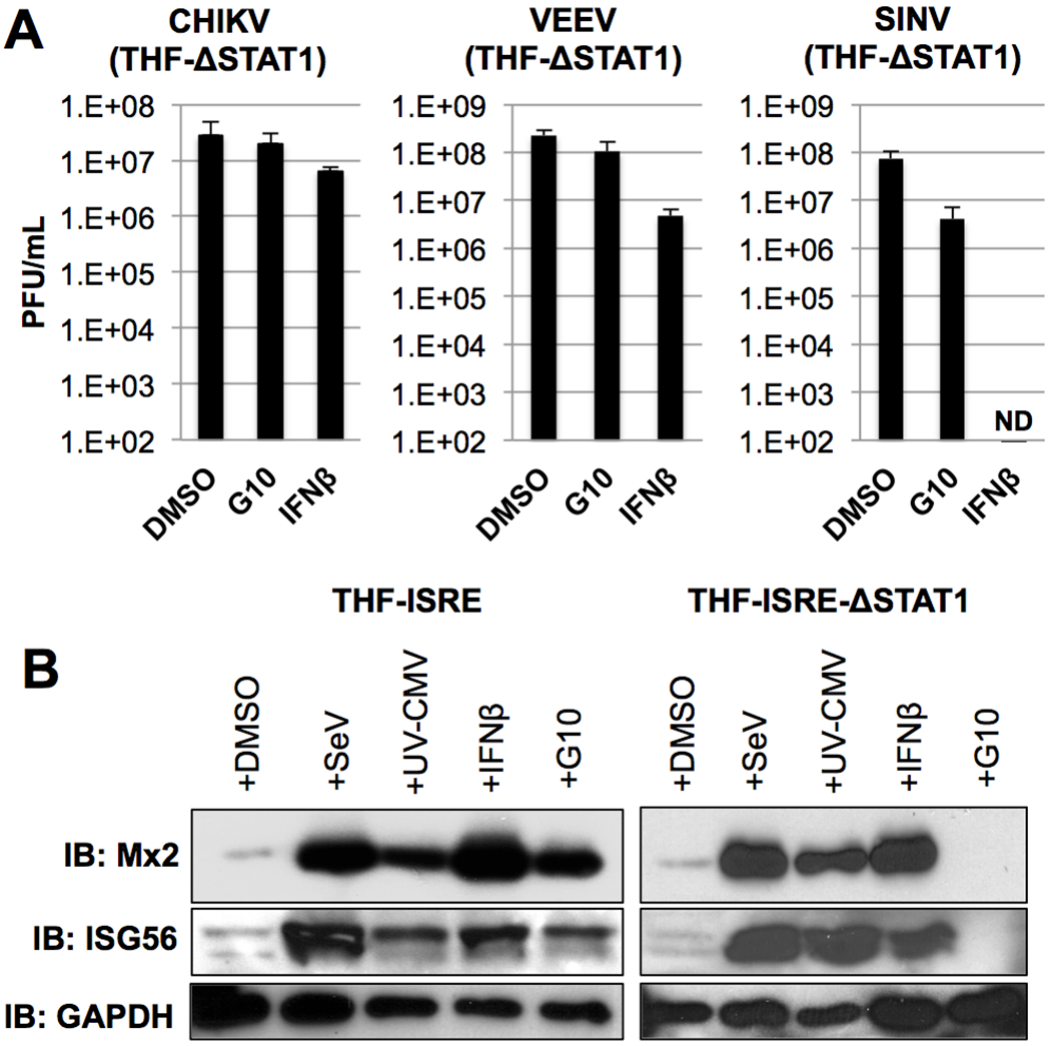
Abrogation of G10-Mediated Antiviral Activity and ISG Expression in Cells Lacking STAT1. (A) Average media titers ±SD of CHIKV, VEEV, and SINV at 24h (VEEV) or 48h (CHIKV, SINV) obtained from THF-ISRE-ΔSTAT1 cells treated with 1% DMSO, 100μM G10, or 1000U/mL IFNβ as indicated. Infections were performed in triplicate. (B) Synthesis of Mx2 and ISG56 proteins in THF-ISRE and THF-ISRE-ΔSTAT1 following 24h exposure to SeV, UV-CMV, 1000U/mL IFNβ or 100μM G10 as indicated.

G10 exposure elicits secretion of bioactive IFN in cells that contain STING and IRF3 (Fig 7C) but also the expression of two classes of ISGs: Those that are induced by either IRF3- or IFN-dependent signaling (ISG15, ISG54, ISG56, Viperin) and those whose expression is activated only by IFN-mediated Jak/STAT signaling (Mx2, OAS). Since many IRF3-dependent genes are direct antiviral effectors (see [79,82]) we asked whether the G10-mediated anti-Alphaviral cellular state we observe could be elicited in the absence of canonical STAT1-mediated, IFNα/β-induced signaling by examining replication of CHIKV, VEEV, and SINV on cells lacking STAT1. As shown in Fig 8A replication of these viruses in cells treated with G10 from which STAT1 was deleted is similar to that seen in the presence of DMSO alone. Intriguingly, IFNβ appears to induce detectable yet significant (in the case of VEEV and CHIKV) and profound (in the case of SINV) antiviral effects even in the absence of STAT1. These data suggest the expression of antiviral effectors that are IFN-induced yet independent of STAT1. Cells lacking STAT1 can still react to IFN-exposure via the activity of STAT2, STAT3, STAT4, or STAT5. In fact, expression of antiviral ISGs (including those examined here) has been detected in the absence of STAT1, likely via STAT2 homodimers [83–85]. In light of these results we examined whether IFN or G10 could stimulate expression of either class of ISG in the absence of STAT1. As shown in Fig 8B SeV, UV-CMV, IFNβ, and G10 were all capable of triggering synthesis of Mx2 and ISG56 proteins. Surprisingly, however, in STAT1-deficient cells Mx2 and ISG56 proteins were still detectable following exposure to SeV, UV-CMV, and IFNβ but not G10. These results are consistent with previous studies showing that some IFN-responsive antiviral effectors are inducible in the absence of STAT1 [83–87]

### G10 triggers IRF3-dependent activity with less potency than 2’3’-cGAMP

**Fig 9:**
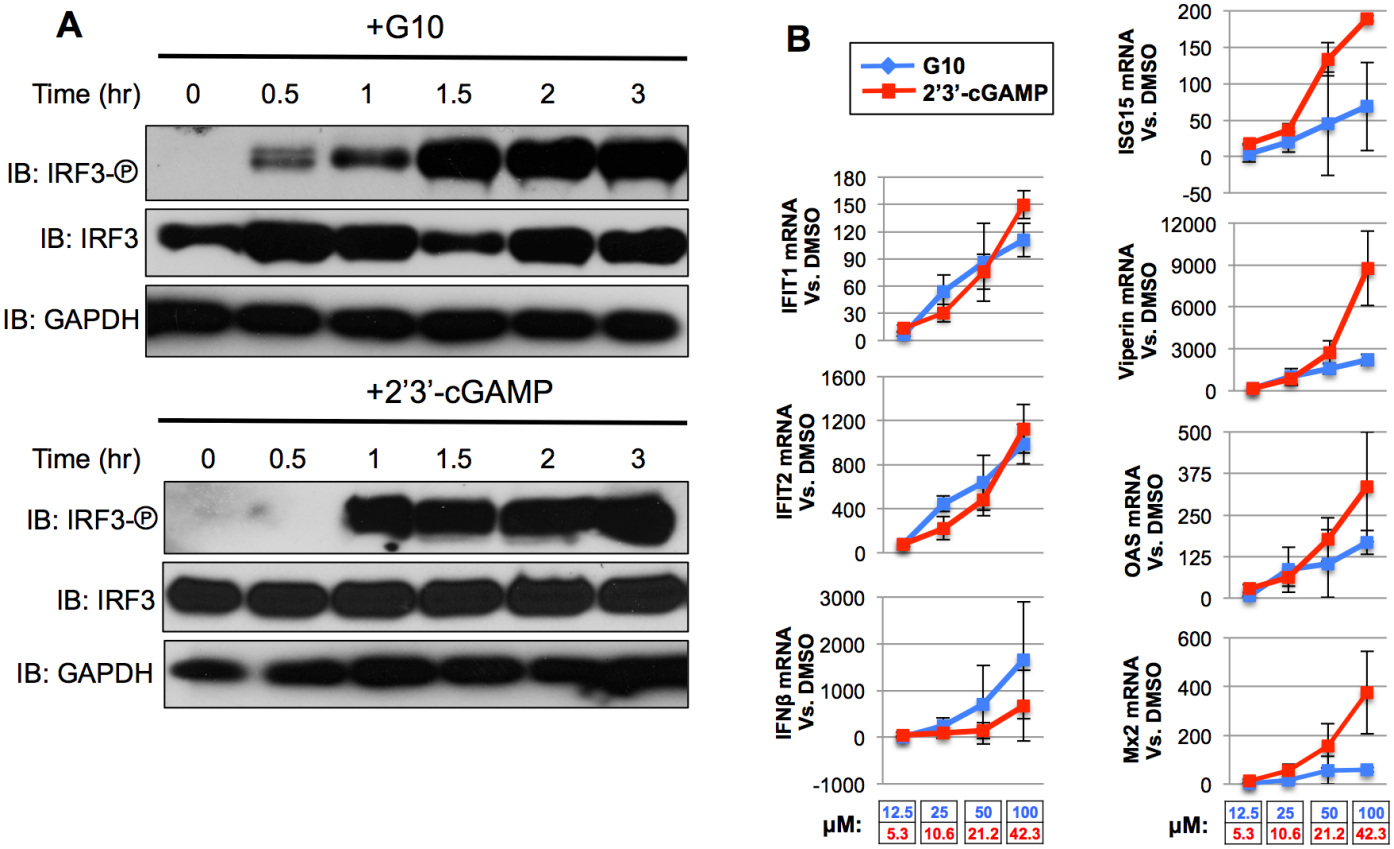
Comparative Kinetics and Dose-Dependence of Innate Immune Activation by G10 and 2’3’-cGAMP. (A) Immunoblot of lysates from THF-ISRE cells following exposure to G10 (100μM) or transfected 2’3’-cGAMP (42.3μM) for indicated time showing phosphorylation status of IRF3 S386, total IRF3, and GAPDH. (B) mRNA synthesis of indicated genes in THF following 8h exposure to indicated concentration of G10 (blue) or 2’3’-cGAMP (red). Indicated values represent average mRNA fold change ±SD from duplicate experiments relative to cells exposed to 1% DMSO.

Our observations indicating that STING is required for G10-mediated induction of IRF3-dependent cellular activity led us to examine how the response to the molecule compares to that of a canonical STING ligand such as 2’3’-cGAMP. For this we examined the kinetics of IRF3 phosphorylation and levels of IRF3-dependent gene induction following exposure to each of the molecules. As shown in Fig 9A phosphorylation of IRF3 S386 is detectable at 30m post exposure to G10 and at 1h post-exposure to 2’3’-cGAMP. It is important to note that 2’3’-cGAMP was transfected into THF and this may affect timing of observable IRF3 phosphorylation. We next examined the transcriptional induction of IRF3- and IFN-dependent genes in response to a range of G10 and 2’3’-cGAMP concentrations. Fig 9B illustrates that 2’3’-cGAMP elicits stronger mRNA synthesis of IFIT1, IFIT2, ISG15, Viperin, OAS, and Mx2 at lower concentrations than G10. Oddly, transcription of IFNβ appeared to show a somewhat different pattern with dose-dependent induction being roughly similar between the molecules. These results indicate that innate immune reactivity may occur more quickly in cells exposed directly to G10 relative to 2’3’-cGAMP (following transfection) but that higher concentrations of the molecule are needed to trigger similar levels of IRF3/IFN-dependent gene expression.

### G10 induces innate antiviral mRNA expression in primary human cells

**Fig 10:**
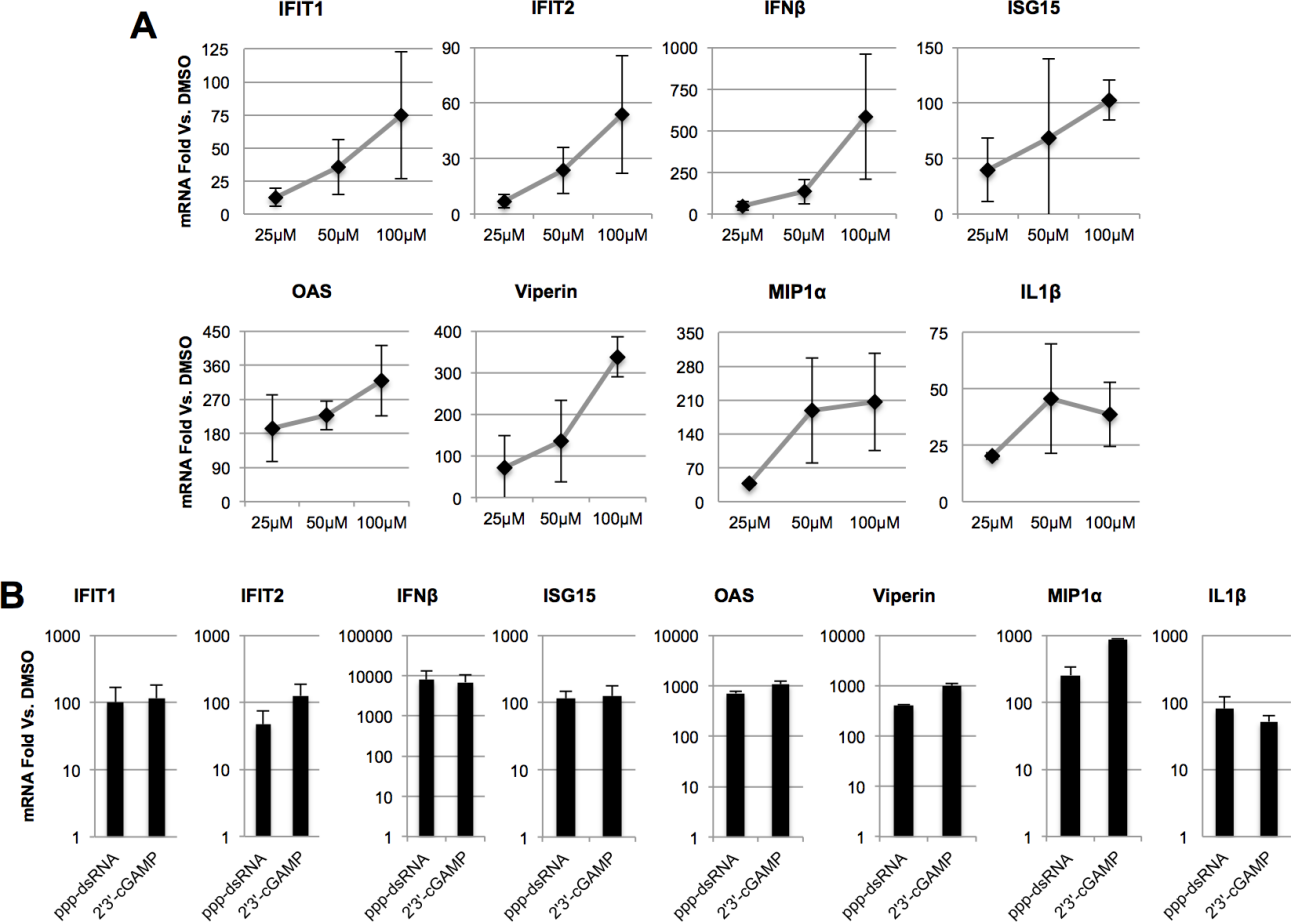
G10-mediated Induction of mRNA in PBMCs. mRNA synthesis of indicated genes in human peripheral blood mononuclear cells (PBMC) following 8h exposure to indicated concentration of G10 (A) or ppp-dsRNA (12.5μg/mL) or 2’3’-cGAMP (28μM) (B). Indicated values represent average mRNA fold change ±SD from duplicate experiments relative to cells exposed to 1% DMSO.

To this point our examination of G10-mediated innate activation has focused on human fibroblasts that, while not strictly immortalized, are life-extended through the introduction of telomerase reverse transcriptase. To determine whether G10 is immunostimulatory to a similar degree in more physiologically relevant primary cell types we examined the transcription of IRF3-, IFN-, and NF-κB-dependent genes in human peripheral blood mononuclear cells (PBMCs). As shown in Fig 10, G10 triggered expression of IFIT1, IFIT2, IFNβ, ISG15, OAS, and Viperin to degrees that were proportional to compound concentration. Interestingly, NF-κB-dependent genes MIP1α and IL1β were also transcriptionally induced by G10, a phenomenon not observed in THF cells (Fig 2). Respective IPS-1- and STING-inducing control stimuli ppp-dsRNA and 2’3’-cGAMP were similarly capable of activating transcription of these genes in these cells (Fig 10B). As shown in S5 Fig primary human umbilical microvascular endothelial cells also responded to G10 exposure by expressing IRF3-, IFN-, and NF-κB-dependent genes. These results clearly demonstrate that innate immune induction by G10 is not an effect specific to the cell model and suggests that in vivo stimulation by G10 is likely to be feasible.

### STING agonism inhibits CHIKV replication in vivo

**Fig 11:**
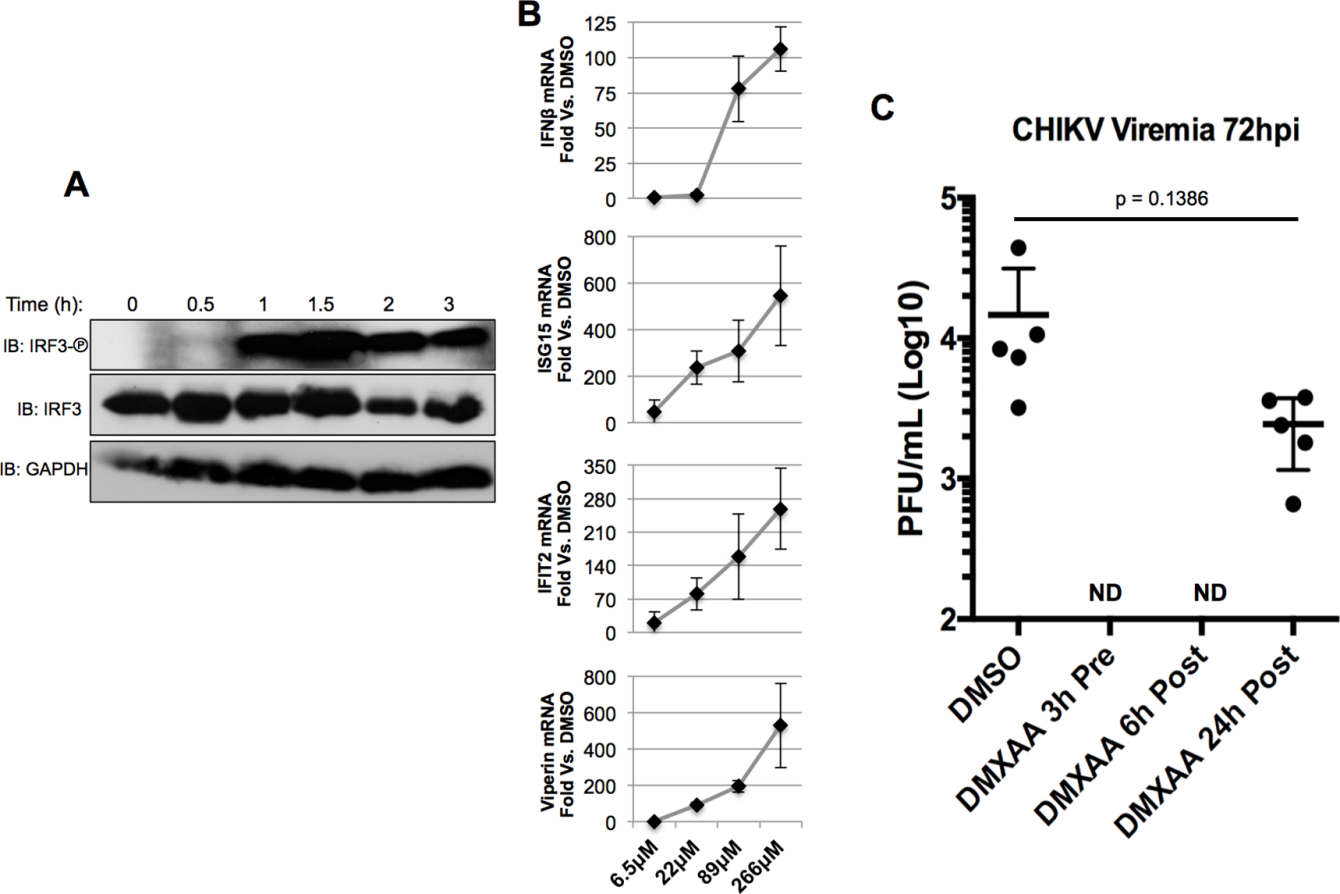
Innate Immune Activation by and Antiviral Activity of DMXAA. **A.** Immunoblot of lysates from murine RAW264.7 cells following exposure to 100μM DMXAA for indicated time showing phosphorylation status of IRF3 S386, total IRF3, and GAPDH. (B) mRNA synthesis of indicated genes in RAW264.7 cells following 8h exposure to indicated concentration of DMXAA. Values represent average mRNA fold change ±SD from duplicate experiments relative to cells exposed to 1% DMSO. (B) Levels of serum-associated CHIKV at 72h post infection. Five mice per group were treated with DMSO alone or DMXAA at 3h pre- or 6h or 24h post inoculation as indicated.

While G10-induced innate immune activation is observed in multiple human cell types, we were unable to detect similar activity in murine myeloid-derived RAW264.7 cells (S6 Fig). Multiple molecular analogs of G10 were thus constructed in an attempt to identify one that is active in both human and mouse cells. While this allowed characterization of essential and nonessential moieties within the molecule (S7 Fig), no derivatives were identified that were active across species. However, 5,6-dimethylxanthenone-4-acetic acid (DMXAA) is a small molecule that has been examined extensively and shown to trigger STING-dependent IRF3 and interferon activity in murine but not human cells ([40,41] and S3 Fig and S6 Fig). As such, the compound has been explored for multiple immunotherapeutic uses including anti-angiogenesis [88], vaccine adjuvanticity [89], anti-tumor immunology [38] and antiviral activity [32,33,90]. We therefore explored patterns of DMXAA-stimulated innate immune activation in murine cells to evaluate their resemblance with those induced by G10 in human cells. Fig 11A illustrates that DMXAA-induced IRF3 phosphorylation in RAW264.7 macrophage-like cells is detectable by 1h post-treatment. Furthermore, DMXAA also elicits dose-dependent transcription of key innate antiviral genes IFNβ, ISG15, IFIT2, and Viperin in a manner similar to that observed for G10 in human cells (Fig 11B). The physiological effects of DMXAA thus appear to resemble those we observe for G10. Fortunately, wild type mice represent a commonly used model of CHIKV infection that manifests viremia, pathogenesis, and immune responses resembling those seen in human patients [46,91–93]. We therefore decided to use this model to ask whether artificial stimulation of STING-dependent signaling was sufficient to block CHIKV replication in vivo.

DMSO or DMXAA (25mg/kg) were administered to mice intraperitoneally at 3h pre-infection with CHIKV (1000 PFU). As shown in Fig 11 treatment with DMSO resulted in an average titer of 1.47 x 10^4^ PFU/mL serum at 72h post infection. In contrast, serum-associated virus was undetectable by plaque assay in mice pre-treated with DMXAA. We next examined whether viral titers were influenced by DMXAA given post-inoculation. For this we administered DMXAA at 6h and 24h post CHIKV infection. Fig 11 illustrates that virus was still undetectable in serum from mice treated at 6h post infection. However, mice treated at 24h post infection showed viral titers that were diminished relative to DMSO-treated animals but this difference was not statistically significant (p = 0.1386). Overall these data indicate that artificial stimulation of STING-dependent signaling in vivo within an appropriate temporal window can block CHIKV replication. It is likely that the antiviral efficacy of STING activation will diminish as time after acute viral replication increases and viral innate evasion phenotypes appear. It is also possible that recurrent STING activation could have an effect on persistence of CHIKV genomic RNA in joint tissues [94] and we are currently examining this possibility.

## Discussion

Pharmacologic activation of STING-dependent signaling represents a potentially high-impact therapeutic strategy with applications in diverse clinical areas such as broad-spectrum antivirals, vaccine adjuvants, vascular disruption, and antitumor immunology. This is represented by multiple successes of the utilization of this approach in mouse models of virus infection [29,32,33,90,95], enhancement of vaccine immunogenicity [89,96–98], immune-mediated tumor necrosis [36,38,99], and inhibition of solid tumor angiogenesis [100,101]. Unfortunately, synthetic small molecules identified thus far have only exhibited suitable efficacy in mouse models due to their strict specificity for the murine STING ortholog [39–41]. Here we employed high-throughput screening to identify a novel compound (G10) capable of triggering IRF3/IFN-dependent responses and subsequently blocking replication of CHIKV, VEEV, and SINV in human cells. Follow-up work seeking to pinpoint cellular targets essential to the phenotypic responses utilized a reverse genetics approach by way of CRISPR/Cas9-mediated genome editing. This enabled identification of the STING protein as required for G10’s biological activity thus indicating that the compound is the first described human-specific synthetic small molecule STING agonist.

G10 triggers innate immune responses that involve expression of IRF3-dependent genes including type I and III interferons. This was observed in telomerized foreskin fibroblasts as well as primary cells such as PBMCs and endothelial cells. Unexpectedly, however, G10 did not induce expression of genes associated with the activity of NF-κB in fibroblasts even though such genes were induced in PBMCs and endothelial cells. Given the central role of NF-κB in generation of pro-inflammatory states that can lead to pathogenic consequences, especially under chronic circumstances, (reviewed in [102,103]), it is perhaps desirable that the activity of G10 is more transcriptionally focused to IRF3-dependent responses in certain cell types. It is also interesting that G10 induces type I IFN synthesis in the absence of detectable NF-κB activity given the reported requirement of the transcription factor for this process [104–107]. Activation of noncanonical NF-κB subunits may play a role in this case. Undertaking a more thorough molecular investigation of NF-κB subunit activation (e.g. nuclear localization, phosphorylation, DNA binding) will be required to understand this with greater clarity. Importantly, G10 induced the phosphorylation of IRF3 and the protein’s deletion led to elimination of reporter gene transcription as well as the compound’s anti-Alphaviral activity. As such the innate biological effects of G10 examined here require IRF3-driven gene expression.

Deletion of the adaptor molecule IPS-1/MAVS did not eliminate G10-induced IRF3 phosphorylation or affect the molecule’s antiviral effect (Fig 5). Furthermore, G10-associated transcription of IRF3-dependent genes was also intact in the absence of IPS-1 (Fig 6). In light of these results, it is intriguing that G10-induced IFN secretion was diminished following IPS-1 deletion (Fig 7). This result is consistent with data showing interaction between IPS-1 and STING during RIG-I-mediated stimulation (reviewed in [54]) although whether STING strictly requires this interaction for full signaling has not been shown. Nevertheless, IPS-1 does not appear to play a substantial role in G10-mediated anti-Alphaviral activity and thus upstream IPS-1-dependent PRRs such as RIG-I and MDA5 are unlikely to be engaged by G10 or relevant for these effects.

Our results clearly establish an essential role for the signaling molecule STING. Deletion of the STING protein resulted in complete inactivation of G10-mediated IRF3 phosphorylation, IRF3-dependent transcription, IFN secretion, and antiviral activity (Figs 6 and 7). These results plainly signify that STING-dependent function(s) are necessary for the innate phenotypic response elicited by G10. Whether G10 represents a directly binding and activating synthetic ligand of human STING (as are DMXAA and CMA for mouse STING) was examined using thermal shift assays of purified protein. These revealed no increase in the thermal stability of STING-CTD in the presence of G10 as was seen for 2’3’-cGAMP (S3 Fig). If the molecule bound directly to STING-CTD we would expect a net increase in the melting temperature of the protein dimers [66] as observed when a bona fide ligands such as 2’3’-cGAMP is co-incubated. Additionally, the inability of G10 to induce IRF3-dependent transcription in THP-1 cells (S4 Fig) is also not consistent with the molecule behaving as a direct ligand since these cells express biologically functional STING, as described in numerous studies [16,70,72–77]. The identity of the protein or factor engaged by G10 that ultimately stimulates the STING-dependent response is currently under investigation. IRF3-activating, STING-dependent sensors such as IFI16, DDX41, and cGAS are also present and functional in THP-1 cells [17,73–75,78,108]. As such, it is probable that G10 either engages an alternative STING-dependent PRR such as ZBP1/DAI [18] or IFI203 [17] or an as yet uncharacterized STING-dependent PRR [71]. ZBP1/DAI is a particularly attractive target since we have previously shown it to be expressed and biologically active in THF cells [18]. We are currently addressing this question using CRISPR/Cas9-mediated genome editing and ZBP1/DAI and IFI203 overexpression studies.

Given that G10 stimulates innate cellular effects that require STING we decided to compare the dose dependence of these effects to 2’3’-cGAMP, an established STING ligand. Our results indicate that while G10 may trigger earlier IRF3 phosphorylation than 2’3’-cGAMP, perhaps due to its smaller size and cell permeability, it triggers levels of IRF3-dependent gene expression with overall less potency than 2’3’-cGAMP (Fig 9). More precisely, 2’3’-cGAMP induces higher levels of IRF3- and IFN-dependent mRNA expression at lower concentrations than G10. It would be interesting to establish whether these dissimilarities are causally linked to differences in the molecules’ cellular targets, especially whether their proximity in the signaling cascade to IRF3-directed kinases is important. Alternatively, differences in physico-chemical properties between the molecules and how those relate to solubility and permeability may also impact stimulatory potency [109].

G10 induces synthesis and secretion of bioactive type I and III IFNs and generates an antiviral state in fibroblast cells positive for STING, IRF3, and STAT1 proteins. Based on these results our model for the elicitation of anti-Alphaviral activity by G10 first involves STING-dependent induction of IRF3 followed by IRF3-mediated synthesis and secretion of type I and III IFNs and subsequent IFN-stimulated, STAT1-dependent expression of antiviral effectors. Detection of STAT1-independent ISG expression in response to IFN exposure and IFN-inducing stimuli was unexpected but not unprecedented and has been reported in multiple studies [83–85,110,111]. Blaszcyk and colleagues attribute this to IFN-induced transcriptional complexes composed of IRF9 and STAT2 homodimers [84] although homo- and heterodimers of other Jak/Tyk2-phosphorylated STAT proteins may also play roles (reviewed in [112]). Interestingly, IFNβ was able to stimulate some antiviral activity in cells lacking STAT1 and to a degree that varied between viruses with SINV replication being undetectable. The full assortment of STAT1-independent ISGs expressed cannot be inferred from two proteins (Mx2 and ISG56) and as such the differential susceptibilities of CHIKV, VEEV, and SINV to ISG-encoded proteins in general cannot be known based on these results. Yet it is clear that SINV is highly sensitive to STAT1-independent ISGs relative to the other Alphaviruses. Intriguingly, however, while other IRF3-activating, IFN-inducing stimuli were capable of triggering expression of Mx2 and ISG56 in the absence of STAT1, G10 was not. This likely explains the reliance on STAT1 of G10-mediated anti-Alphaviral activity. Why this disparity in STAT1-dependence occurs between SeV, UV-CMV, and G10 is not clear. It is possible that each stimulus triggers the secretion of unique signatures of type I and type III IFN subtypes that subsequently elicit distinct gene expression patterns [113,114]. Elucidation of the importance of the various IFN proteins in G10’s antiviral effects will require more detailed examination, for instance by comparative transcriptomics, by using subtype-specific neutralizing antibodies or reverse genetics via gene editing.

While the majority of our investigation employed fibroblast cells, it is evident that G10 elicited innate immune activation in primary human cells such as PBMC’s (Fig 10) and umbilical endothelial cells (S5 Fig). Unexpectedly, however, induction of NF-κB-dependent transcription by G10 was observed in primary cells but not fibroblasts. Moreover, no G10-induced IRF3-dependent activity was detected in THP-1 cells (S4 Fig). These disparities may be related to differences in cell type-specific expression of PRRs or innate signaling molecules, especially between stromal versus myeloid-derived cells [115] and between transformed and untransformed cells ([116] and references therein). Understanding the biological bases for these divergent effects will require additional experimentation. However, demonstrating efficacy of G10 on primary human cells is obviously crucial to assessing the therapeutic potential of the compound.

Unfortunately G10 was unable to stimulate similar activation in murine cells. As such, evaluating the in vivo efficacy of G10 using a well-established mouse model of Alphavirus (CHIKV) infection was not directly practical. Yet IFN-inducing STING agonists (e.g. DMXAA, CMA) have been described that are murine specific [39–41]. We therefore examined whether DMXAA triggers IRF3 activation and IRF3-dependent gene induction in a manner comparable to G10. While comparisons of absolute responses are complicated by the fact that different species, cell types, and reagents are employed, DMXAA does trigger rapid IRF3 phosphorylation and dose-dependent IFNβ and ISG transcription in mouse cells (Fig 11) as does G10 in humans cells. In light of this we used the mouse model of acute CHIKV infection to ask whether activation of the STING pathway is feasible as an in vivo anti-Alphaviral strategy. We demonstrate that DMXAA clearly blocks viremia but that this is related to the timing of administration with early (3h pre- or 6h post-infection) being more effective than late (24h post-infection) treatment. It is probable that these kinetics correlate with the appearance of CHIKV-encoded IFN/ISG evasion phenotypes and as such STING-dependent antiviral efficacy diminishes with time post infection. However, whether STING activation represents an effective approach for diminishing persistent (e.g. >6 weeks) CHIKV infection [94,117] is an enticing possibility that warrants examination since this could lead to alleviation of chronic virus-associated arthralgia and is currently being examined in our laboratory.

In summary we have identified a novel synthetic small molecule capable of inducing expression of type I and III IFNs as well as IFN-dependent antiviral effector genes. Using a reverse genetics approach based on CRISPR/Cas9-mediated genome editing to identify cellular targets of the molecule we shown that this effect requires STING, IRF3, and STAT1 proteins. These molecules are likewise essential to the ability of G10 to elicit a cellular state refractory to replication of Alphavirus species. Furthermore, given the pivotal role of STING we also show that pharmacologic activation of the molecule represents an effective anti-Alphaviral strategy in vivo. Given the demonstrated role of STING pathway stimulation in numerous immunological processes, it is being pursued as a therapeutic target for many diseases. Our work demonstrates the feasibility of identifying molecules that activate STING-dependent signaling and yield therapeutic outcomes as well as a strategy for characterizing cellular effects and essential modulatory proteins via genome editing.

## Materials and Methods

### Reagents and antibodies

Dimenthyl sulfoxide (DMSO) was obtained from Thermo-Fisher. Puromycin was obtained from Clontech and used at 3μg/mL in cell culture medium. Lipopolysaccharide (LPS) and polybrene were obtained from Sigma-Aldrich. Human recombinant IFNβ and tumor necrosis factor α (TNFα) were obtained from PBL. ONE-Glo cell lysis/luciferin reagent was obtained from Promega. Lucia luciferin reagent was obtained from Invivogen. Lipofectamine LTX was obtained from Life Technologies. Poly(I:C) was obtained from Amersham (27–4729). 2’3’-cGAMP and ppp-dsRNA were purchased from Invivogen (tlrl-cga23 and tlrl-3prna, respectively). Unless otherwise indicated cells were exposed to ppp-dsRNA at 12.5μg/mL based on a dose response of innate immune activity performed on THF cells. Stocks of G10 were purchased from ChemDiv. DMXAA was purchased from ApexBio. Antibodies used against the following antigens are indicated in parentheses: GAPDH (Santa Cruz SC-51906); STAT1 (Santa Cruz SC-346) IRF3 (Santa Cruz SC-9082); human S386 phospho-IRF3 (Epitomics 2562–1); mouse S396 phospho-IRF3 (cell Signaling 4947); STING (Cell Signaling 3337); IPS-1 (Bethyl A300-782A); IFIT1/ISG56 (Thermo Fisher PA3 848); and Mx2 (Sigma HPA030235).

### Cell and virus culture

Human foreskin fibroblasts originally obtained from the American Type Culture Collection were stably transduced with constitutively expressed human telomerase reverse transcriptase and the IRF3/IFN-responsive pGreenFire-ISRE lentivector and were maintained in DMEM containing 10% fetal calf serum (FCS) and antibiotics as described previously [52]. Vero, BHK-21, and C6/36 cells were obtained from Alec Hirsch (Oregon Health and Science University) and were grown as described [49]. RAW264.7 cells were obtained from Jay Nelson (Oregon Health and Science University) and transduced with a lentivector that contains firefly luciferase under the control of the type I IFN responsive element obtained from SA Biosciences. THP1-ISG-Lucia cells were obtained from Invivogen and maintained in RPMI containing 10% FCS and antibiotics. These cells were differentiated in 100nM phorbol 12-myristate 13-acetate (PMA) for 24h before stimulation. Human peripheral blood mononuclear cells were obtained from StemCell Technologies and maintained in RPMI containing 10% FCS and antibiotics. Human umbilical microvascular endothelial cells were obtained from Patrizia Caposio (Oregon Health and Science University) and maintained as described [118]. All cells were grown at 37°C and 5% CO_2_. Sendai virus (SeV) was obtained from Charles River Laboratories and used at 16 HA units/mL. Cytomegalovirus was grown, titered, UV-inactivated, and exposed to cells as described previously [51,52]. West Nile Virus (WNV) was obtained from Alec Hirsch (Oregon Health and Science University) and used as previously described [119]. Vaccinia Virus (VACV) strain Western Reserve was obtained from Klaus Früh (Oregon Health and Science University) and used as previously described [120]. Sindbis virus (SINV) strain Ar-339 was obtained from ATCC. Venezeulan encephalitis virus (VEEV) strain TC83 and Chikungunya virus (CHIKV) strain MH56 were obtained from Michael Diamond (Washington University). CHIKV was derived from an infectious clone as follows. RNA was transcribed from the linearized clone using the T7 mMessage mMachine kit (Ambion) and transfected using Lipofectamine LTX into BHK-21 cells. Resultant virus was propagated in C6/36-insect cells for 48h to produce high titer viral stocks after pelleting through a 20% sucrose cushion by ultracentrifugation (22,000 rpm, 825206g for 1.5 hrs). In all cases infectious virus was quantified by serial dilution plaque assays on Vero cells with a carboxymethylcellulose overlay. Unless otherwise indicated experimental infections were carried out in triplicate using a multiplicity of infection (MOI) of 1 plaque forming unit (PFU) per cell. Cell viability was examined by quantitating ATP using the Cell Titer GLO assay according to the manufacturer’s instructions (Promega).

### Immunoblotting

Sodium dodecyl sulfate-polyacrylamide gel electrophoresis (SDS-PAGE) immunoblots were performed as follows. After trypsinization and cell pelleting at 2,000 x g for 10 min. whole-cell lysates were harvested in 2% SDS lysis buffer (50 mM Tris-HCl, 20% glycerol). Lysates were electrophoresed in 8% polyacrylamide gels and transferred onto polyvinylidene difluoride membranes (Millipore) using semidry transfer at 400 mA for 1h. The blots were blocked at room temperature for 2h or overnight using 10% nonfat milk in 1x PBS containing 0.1% Tween 20. The blots were exposed to primary antibody in 5% nonfat milk in 1x PBS containing 0.1% Tween 20 for 18 h at 4°C. The blots were then washed in 1x PBS containing 0.1% Tween 20 for 20, 15, and 5 min, followed by deionized water for 5 min. A 1h exposure to horseradish peroxidase-conjugated secondary antibodies and subsequent washes were performed as described for the primary antibodies. The antibody was visualized using enhanced chemilumi- nescence (Pierce).

### RNA isolation and semiquantitative reverse transcription-PCR (RT-PCR)

Total RNA was isolated from cells and DNased using a DNA Free RNA Isolation kit according to the manufacturer’s protocol (Zymo Research) and quantified by UV spectrometry. Single-stranded cDNA for use as a PCR template was made from total RNA using random hexamers to prime first-strand synthesis by Superscript III reverse transcriptase (Life Technologies) as described in the manufacturer’s protocol. Comparison of mRNA expression between samples (e.g., treated versus untreated) was performed using semiquantitative real-time RT-PCR (qPCR) with the Applied Biosystems sequence detection system according to the ΔΔCT method [121]. For IFNβ, IFNλ1, and mouse and human GAPDH (housekeeping gene) pre-validated PrimeTime FAM qPCR primer/probe sets obtained from IDT were used. For all other genes Maxima SYBR Green qPCR master mix (Thermo Fisher) was used. Primers for human ISG15, ISG56, ISG54, and Viperin were described in [18,51,52,122]. Other human primer sequences were as follows: Mx2-For, 5’-ACTTCAGTTCAGAATGGAG-3’ Mx2-Rev, 5’-TATTCTGTGAAGGCGTCC-3’ OAS-For, 5’- CAGCGCCCCACCAAGCTCAA-3’ OAS-Rev, 5’- TGCTCCCTCGCTCCCAAGCA-3’ IL8-For, 5’-GACTTCCAAGCTGGCCGT-3’ IL8-Rev, 5’-GAATTCTCAGCCCTCTTCA-3’ IL1β-For, 5’- AACAGGCTGCTCTGGGATTCTCTT-3’ IL1β-Rev, 5’- TGAAGGGAAAGAAGGTGCTCAGGT-3’ MIP1α-For, 5’- GCTGCCCTTGCTGTCCTCCTC-3’ MIP1α-Rev, 5’- GGTCAGCACAGACCTGCCGG-3’. Mouse primers were as follows: IFIT2/ISG54-For, 5’-TCCAGCCCCTACAGGATTGA-3’ IFIT2/ISG54-Rev, 5’-TTCGGGTCCTTTTCCAGAGC-3’ IFNβ-For, 5’-CTGGAGCAGCTGAATGGAAAG-3’ IFNβ-Rev, 5’-CTTCTCCGTCATCTCCATAGGG-3’ Viperin-For, 5’-AGCAGGTGTGTGCCTATCAC-3’ Viperin-Rev, 5’-TCAGCCAGCAGAACAGGATG-3.

### Lentivector transduction and CRISPR/Cas9-mediated genome editing

NF-κB-responsive luciferase reporter cells were made using a commercially available replication incompetent lentivirus (Qiagen). Telomerized human fibroblasts were exposed to virus inoculum in the presence of DMEM plus 5μg/mL polybrene and rocked at 37°C for 8h. At two days post inoculation cells were exposed to 3μg/mL puromycin. After cells were fully resistant to puromycin they were verified for responsiveness to NF-κB-inducing stimuli (e.g. TNFα, SeV, LPS). Genome editing using lentivector-mediated delivery of CRISPR/Cas9 components was performed generally as described previously [56]. Briefly, 20nt guide RNA (gRNA) sequences targeting protein-coding regions were inserted into the lentiCRISPRv2 vector (AddGene # 52961). These sequences are as follows. IRF3: GAGGTGACAGCCTTCTACCG; IPS-1: AGTACTTCATTGCGGCACTG; STAT1: AGAACACGAGACCAATGGTG; STING: CCCGTGTCCCAGGGGTCACG. Lentivirus was made by transfecting specific lentiCRISPRv2 plasmid along with packaging (psPAX2; AddGene # 12260) and VSV-G pseudotyping (pMD2.G; Addgene # 12259) plasmids into Lenti-X 293T cells (Clontech) using Lipofectamine-LTX (Life Technologies). Media was harvested at 48h and 72h post transfection, centrifuged (3,000 x *g* for 10 min.) and filtered through a 0.45-μm-pore-size filter to remove cell debris. Subconfluent target cells were exposed to lentivirus for 8h in the presence of 5 μg/mL polybrene. After the cells reached confluence they were split into DMEM plus 10% FCS containing 3μg/mL puromycin. Transduced cells were passaged in the presence of puromycin for 7–10 days before protein knockout was examined by immunoblot. Cells were next serially diluted twice in 96 well plates to obtain oligoclonal lines purified for gene deletion. Protein knockout was additionally verified functionally by measuring phenotypic responsiveness to relevant stimuli as discussed below.

### Luciferase reporter assays

Confluent reporter cells were plated at 20,000 (THF-ISRE) or 100,000 (THP1-ISG-Lucia) cells per well in a white 96 well plate 24h before stimulation. Treatments were performed in quadruplicate in 50μL DMEM plus 2% FCS for 7h unless otherwise indicated. One-GLO lysis/luciferin reagent (Promega) was added at 1:1 to each well and luminescence measured on a Synergy plate reader (BioTek).

### STING protein purification and thermal shift assays

Coding sequences for human STING-C-terminal domain (CTD; AA 137–379) and mouse STING-CTD (AA 137–378) were cloned into pRSET-B vector (Invitrogen) and contained a 6xHIS tag for protein expression in *E*. *Coli* strain BL21 (DE3)pLysS (Promega). Sequences were verified before transforming bacteria, which were then grown in LB media at 37°C until the OD_600_ reached 0.8. Protein expression was induced with 1mM IPTG at 16°C for 18h. After induction, the culture was centrifuged and the pellet resuspended in 50 mM NaH_2_PO_4_, 150 mM NaCl (pH 7.5) and 10% glycerol after which the cells were lysed by sonication. The recombinant soluble STING-CTD was purified by nickel-affinity chromatography (Clontech laboratories) after which it was further purified by gel-filtration chromatography using a HiPrep 16/60 Sephacryl S-100 HR column (GE Healthcare Life Sciences). Protein was eluted in 50 mM NaH_2_PO_4_, 150 mM Nacl (pH 7.5) and the eluted fractions containing STING-CTD concentrated using an Amicon centrifugal filter (10 Kd molecular weight cut-off; Millipore). Aliquots of concentrated STING-CTD were immediately stored at -80°C. For thermal shift assay, 1 μg of recombinant human or mouse STING-CTD was used combined with various concentrations of G10, 2’3’-cGAMP, or DMXAA along SYPRO Orange dye (1:1000 dilution) in a 20μL reaction (in triplicate). A StepOne Plus Real-time PCR system was used to acquire fluorescence. The samples were subjected to a temperature gradient of 25 to 99°C. The melting curves were plotted and *Tm* values determined by fitting the curves to Boltzmann sigmoidal equation using the GraphPad Prism 6 software. Three independent experiments were performed.

### In vivo administration of DMXAA and viral infection

C57Bl/6J mice (5–7 weeks of age, Jackson Laboratories) were housed in cage units in an animal BSL3 facility, fed ad libitum, and cared for under USDA guidelines for laboratory animals. 25mg/kg DMXAA (or DMSO alone) was prepared in 50μL DMSO and injected intraperitoneally. Mice were challenged with 1000 PFU CHIKV via footpad injection in 20μL RPMI under isoflurane-induced anesthesia. Animals were euthanized at 72h post infection by isoflurane overdose. Blood was collected by cardiac puncture and serum viral loads titered on Vero cells in duplicate as described above.

### Ethics statement

All animal procedures were conducted in accordance with and approved by the Oregon Health and Science University Institutional Animal Care and Use Committee (IACUC) under protocol 0913. The Oregon Health and Science University IACUC adheres to the NIH Office of Laboratory Animal Welfare standards (OLAW welfare assurance # A3304-01).

## Supporting Information

S1 Fig ATP-dependent luminescence of THF following 24hr or 48hr exposure to indicated concentrations of G10 or 3% DMSO (0). Values displayed are raw luminescence values averaged from quadruplicate measurements ±SD following 24h or 48h exposure to indicated concentration of G10.
(TIFF)

S2 Fig SINV Growth is Strongly Impaired in WT cells. Average media titers +SD of SINV at 24h or 48h post infection on wild type THF-ISRE cells and at 24h post infection of cells lacking IPS-1 as indicated Infections were performed in triplicate.
(TIF)

S3 Fig Thermal Shift Analysis of Human and Mouse STING-CTD in the presence of G10, DMXAA and 2’3’-cGAMP. **A.** Melting temperature shifts for human STING-CTD in the absence of compound and in the presence of G10 (100μM, 50μM, and 10μM) or 200μM 2’3’-cGAMP. (B) Melting temperature shifts for mouse STING-CTD in the absence of compound and in the presence of G10 (100μM and 10μM), 200μM DMXAA, or 100μM 2’3’-cGAMP. Values presented are averages of triplicate technical replicates and are representative of three independent experiments. (C) Absolute change in hSTING and mSTING melting temperature in the presence of indicated molecule relative to protein alone.
(TIFF)

S4 Fig G10 does not induce IRF3-dependent transcription in promomocytic THP-1 cells. (A) THP1-ISG-Lucia cells differentiated for 24h with 100nM PMA were treated overnight with 1% DMSO, G10 at indicated concentration, UV-CMV, or SeV. Expression of Lucia luciferase was quantitated by measuring luminescence from quadruplicate treatments. Data illustrated are average Lucia fold changes ±SD calculated relative to DMSO-treated cells. (B) mRNA synthesis of indicated genes in differentiated THP-1 cells following 8h exposure to SeV, UV-CMV, or 100μM G10. Indicated values illustrate mRNA fold change and are representative of duplicate experiments relative to untreated cells.
(TIFF)

S5 Fig G10-mediated Induction of mRNA in Primary Human Endothelial Cells. mRNA synthesis of indicated genes in human umbilical microvascular endothelial cells following 8h exposure to UV-CMV or 100μM G10. Indicated values represent average mRNA fold change ±SD from duplicate experiments relative to cells exposed to 1% DMSO.
(TIFF)

S6 Fig G10 does not Induce IFN-Dependent Signaling in Mouse Reporter Cells. Luminescence detected in RAW264.7 cells stably transduced with IFN-dependent LUC (RAW264.7-ISRE) following overnight exposure to 1%DMSO or indicated concentration of DMXAA or G10.
(TIFF)

S7 Fig Ability of G10 analogs to induce luciferase signal in THF-ISRE. Data illustrated are average LUC fold changes ±SD calculated relative to DMSO-treated cells for quadruplicate treatments.
(TIFF)
