# Supplementary figures and images for: Characterization of a Novel Human-Specific STING Agonist that Elicits Antiviral Activity Against Emerging Alphaviruses
Source: PLoS Pathog. 2015 Dec 8;11(12):e1005324. doi: 10.1371/journal.ppat.1005324 (PMC4672893; doi:10.1371/journal.ppat.1005324)

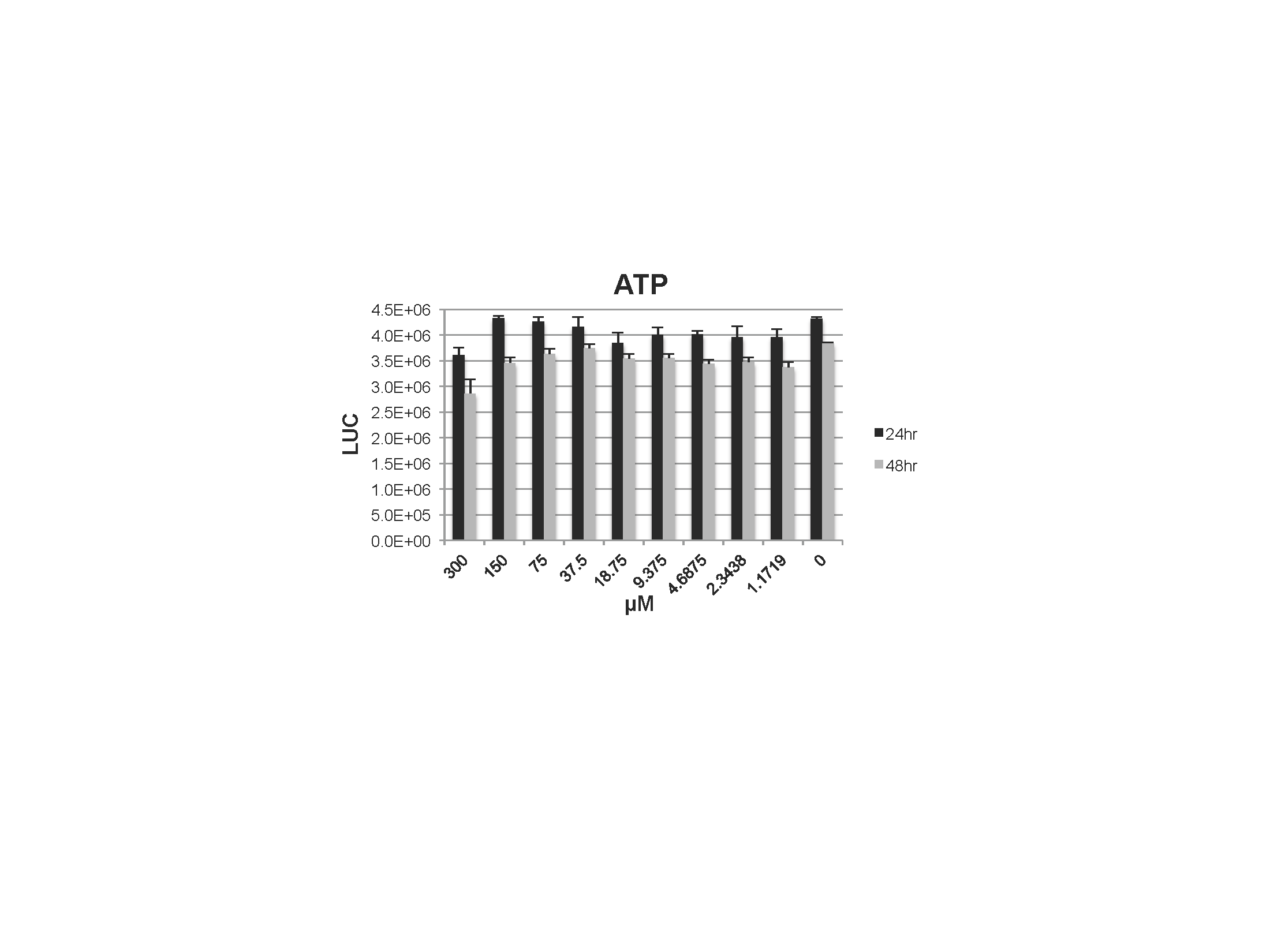

Supplement: S1 Fig — Values displayed are raw luminescence values averaged from quadruplicate measurements ±SD following 24h or 48h exposure to indicated concentration of G10. (TIFF) [file ppat.1005324.s001.tiff]

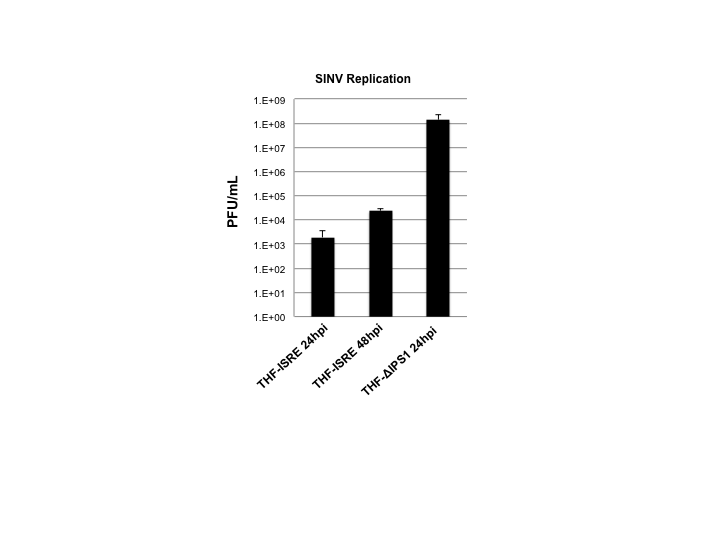

Supplement: S2 Fig — Average media titers +SD of SINV at 24h or 48h post infection on wild type THF-ISRE cells and at 24h post infection of cells lacking IPS-1 as indicated Infections were performed in triplicate. (TIF) [file ppat.1005324.s002.tif]

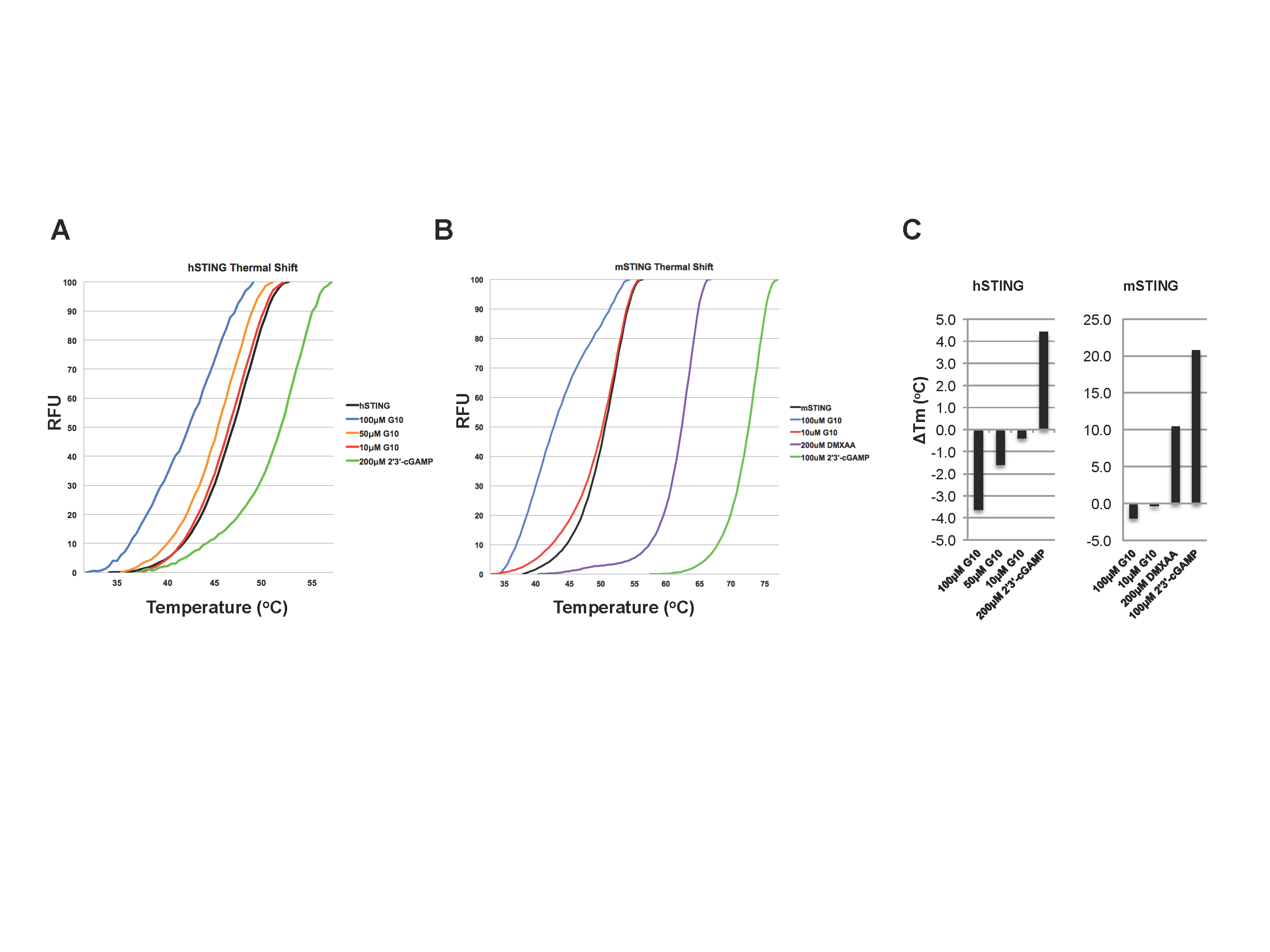

Supplement: S3 Fig — A. Melting temperature shifts for human STING-CTD in the absence of compound and in the presence of G10 (100μM, 50μM, and 10μM) or 200μM 2’3’-cGAMP. (B) Melting temperature shifts for mouse STING-CTD in the absence of compound and in the presence of G10 (100μM and 10μM), 200μM DMXAA, or 100μM 2’3’-cGAMP. Values presented are averages of triplicate technical replicates and are representative of three independent experiments. (C) Absolute change in hSTING and mSTING melting temperature in the presence of indicated molecule relative to protein alone. (TIFF) [file ppat.1005324.s003.tiff]

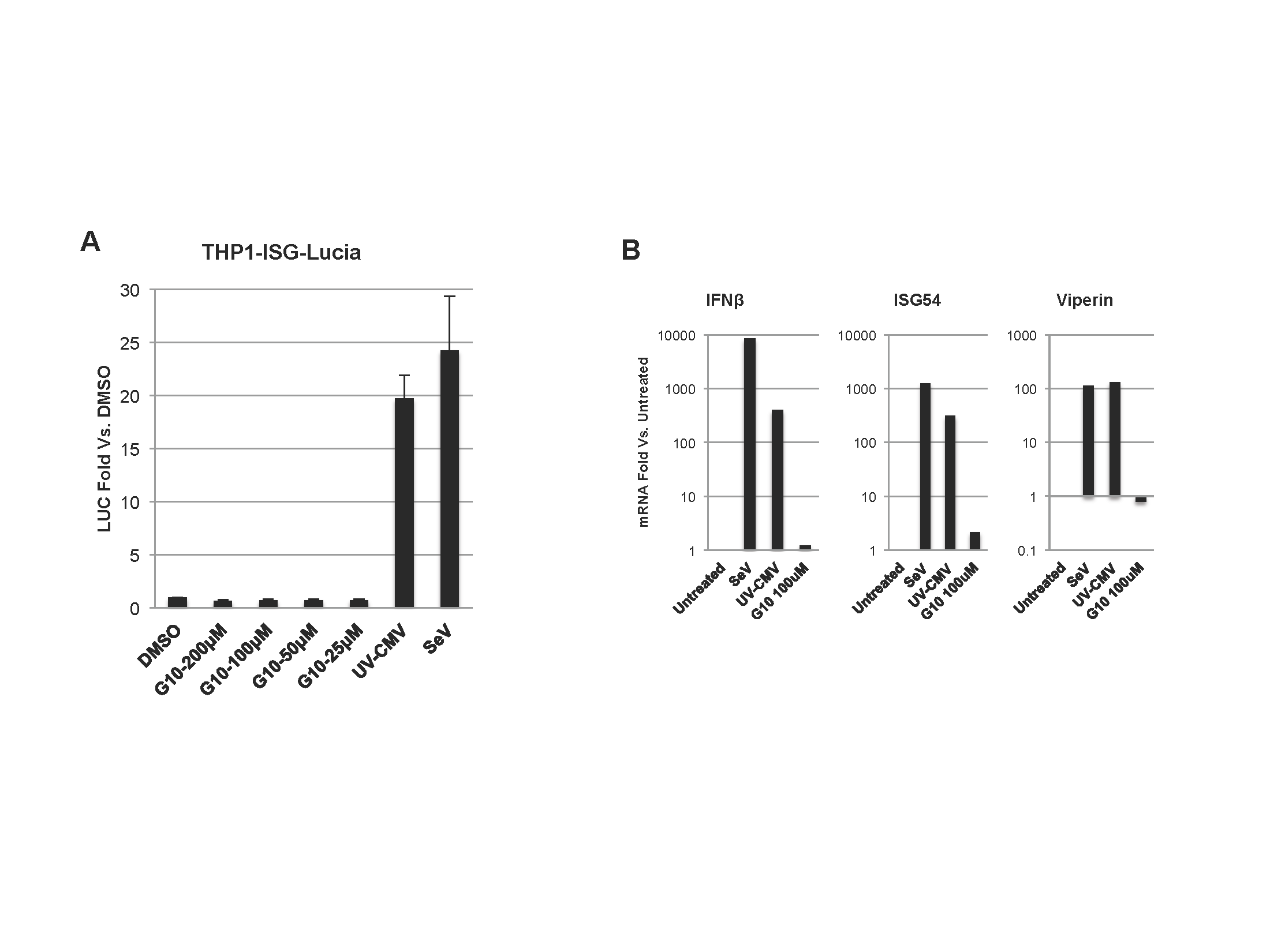

Supplement: S4 Fig — (A) THP1-ISG-Lucia cells differentiated for 24h with 100nM PMA were treated overnight with 1% DMSO, G10 at indicated concentration, UV-CMV, or SeV. Expression of Lucia luciferase was quantitated by measuring luminescence from quadruplicate treatments. Data illustrated are average Lucia fold changes ±SD calculated relative to DMSO-treated cells. (B) mRNA synthesis of indicated genes in differentiated THP-1 cells following 8h exposure to SeV, UV-CMV, or 100μM G10. Indicated values illustrate mRNA fold change and are representative of duplicate experiments relative to untreated cells. (TIFF) [file ppat.1005324.s004.tiff]

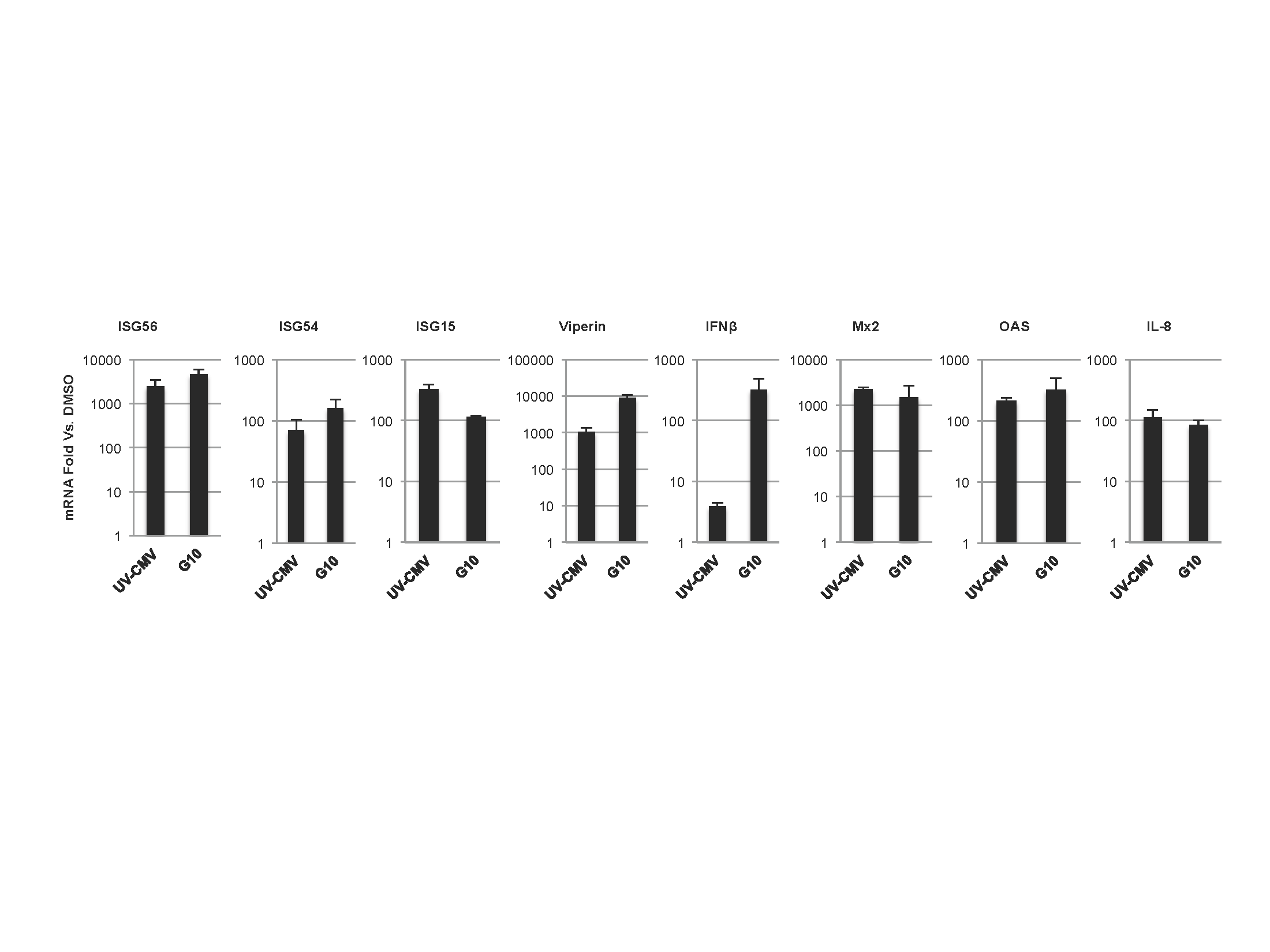

Supplement: S5 Fig — mRNA synthesis of indicated genes in human umbilical microvascular endothelial cells following 8h exposure to UV-CMV or 100μM G10. Indicated values represent average mRNA fold change ±SD from duplicate experiments relative to cells exposed to 1% DMSO. (TIFF) [file ppat.1005324.s005.tiff]

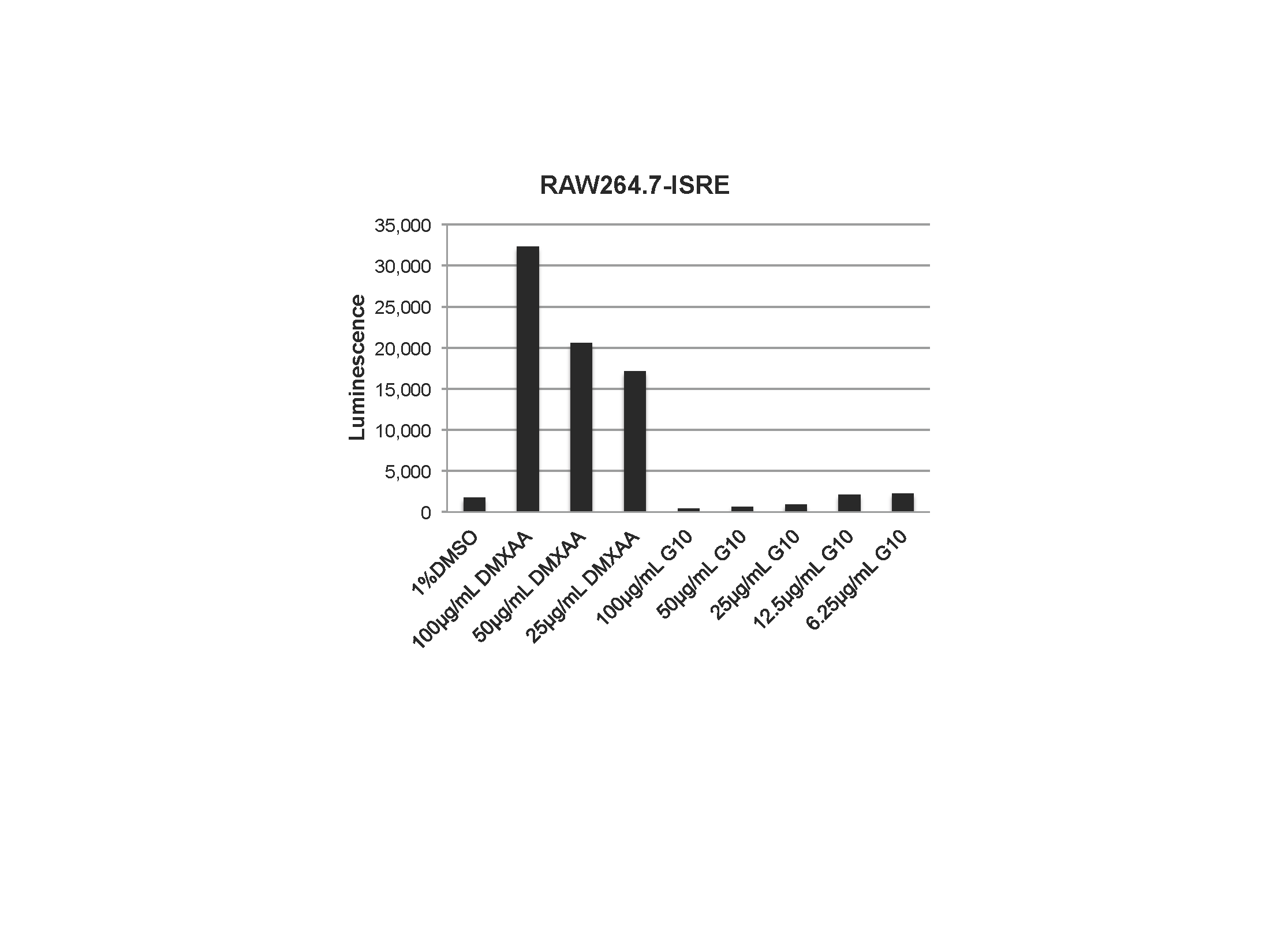

Supplement: S6 Fig — Luminescence detected in RAW264.7 cells stably transduced with IFN-dependent LUC (RAW264.7-ISRE) following overnight exposure to 1%DMSO or indicated concentration of DMXAA or G10. (TIFF) [file ppat.1005324.s006.tiff]

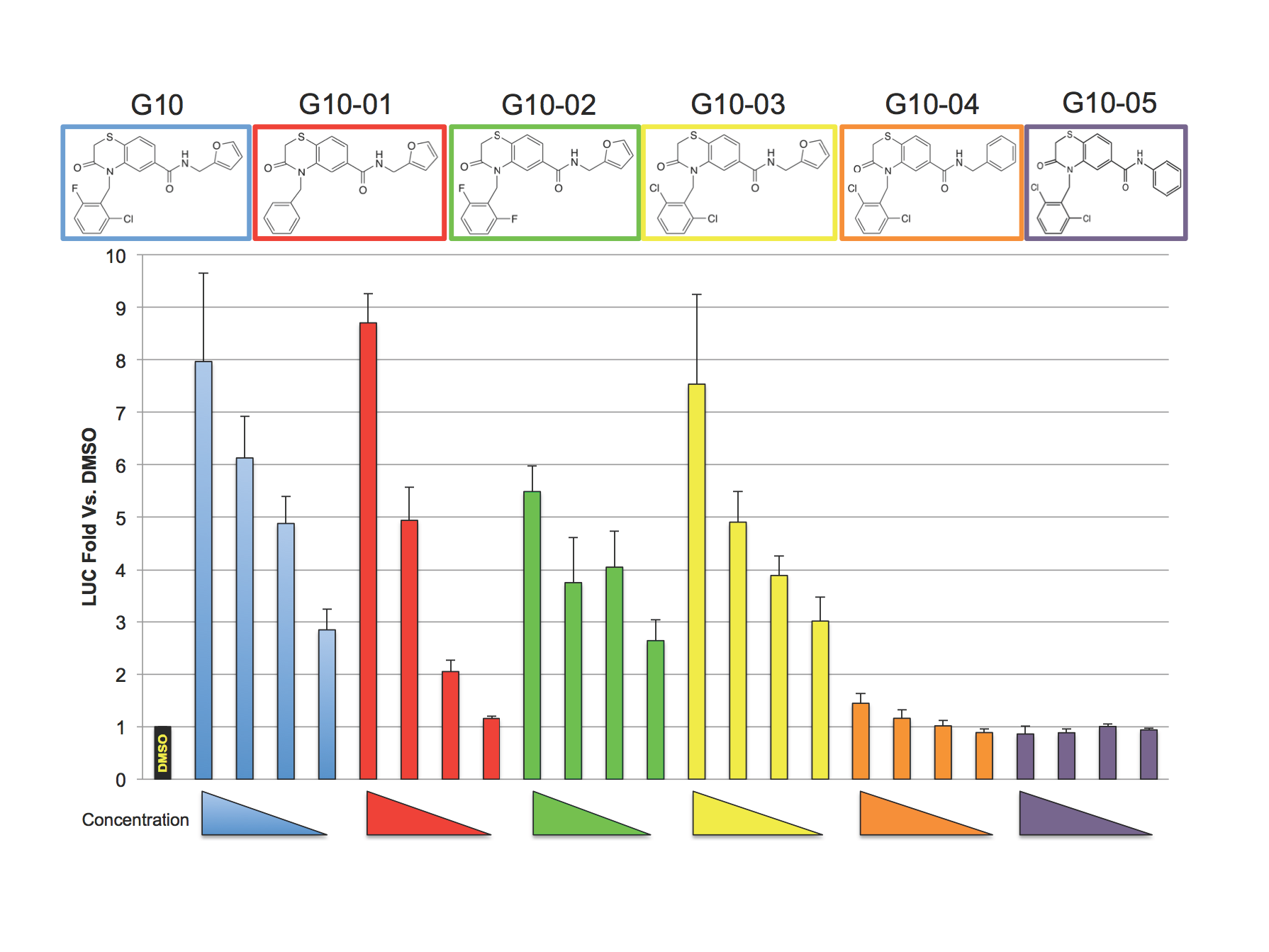

Supplement: S7 Fig — Data illustrated are average LUC fold changes ±SD calculated relative to DMSO-treated cells for quadruplicate treatments. (TIFF) [file ppat.1005324.s007.tiff]
